# Porous Organic Frameworks: Advanced Materials in Analytical Chemistry

**DOI:** 10.1002/advs.201801116

**Published:** 2018-10-17

**Authors:** Shuaihua Zhang, Qian Yang, Chun Wang, Xiliang Luo, Jeonghun Kim, Zhi Wang, Yusuke Yamauchi

**Affiliations:** ^1^ Department of Chemistry College of Science Hebei Agricultural University Baoding 071001 Hebei China; ^2^ Key Laboratory of Sensor Analysis of Tumor Marker (Ministry of Education) Shandong Key Laboratory of Biochemical Analysis, and Key Laboratory of Analytical Chemistry for Life Science in Universities of Shandong College of Chemistry and Molecular Engineering Qingdao University of Science and Technology Qingdao 266042 China; ^3^ School of Chemical Engineering and Australian Institute for Bioengineering and Nanotechnology (AIBN) The University of Queensland Brisbane QLD 4072 Australia; ^4^ International Center for Materials Nanoarchitectonics (MANA) National Institute for Materials Science (NIMS) 1‐1 Namiki Tsukuba Ibaraki 305‐0044 Japan; ^5^ Department of Plant & Environmental New Resources Kyung Hee University 1732 Deogyeong‐daero Giheung‐gu, Yongin‐si Gyeonggi‐do 446‐701 South Korea

**Keywords:** analytical chemistry, chromatographic separation, fluorescence detection, porous organic frameworks, sample preparation

## Abstract

Porous organic frameworks (POFs), a general term for covalent‐organic frameworks (COFs), covalent triazine frameworks (CTFs), porous aromatic frameworks (PAFs), etc., are constructed from organic building monomers with strong covalent bonds and have generated great interest among researchers. The remarkable features, such as large surface areas, permanent porosity, high thermal and chemical stability, and convenient functionalization, promote the great potential of POFs in diverse applications. A critical overview of the important development in the design and synthesis of COFs, CTFs, and PAFs is provided and their state‐of‐the‐art applications in analytical chemistry are discussed. POFs and their functional composites have been explored as advanced materials in “turn‐off” or “turn‐on” fluorescence detection and novel stationary phases for chromatographic separation, as well as a promising adsorbent for sample preparation methods. In addition, the prospects for the synthesis and utilization of POFs in analytical chemistry are also presented. These prospects can offer an outlook and reference for further study of the applications of POFs.

## 1 Introduction

On the microscale and nanoscale, the design, construction, and utilization of advanced porous materials with specific architectures and prominent features has been a topic of research in numerous scientific fields.^1^ Porous materials have been revolutionized, ranging from the traditional inorganic skeleton of zeolites, silica, activated carbon, and hybrid inorganic–organic metal–organic frameworks (MOFs) to pure organic networks of porous organic frameworks (POFs). POFs are ingeniously constructed with organic monomers linked by strong covalent bonds from lightweight, nonmetallic elements (i.e., C, H, N, B, O, and Si). Because of their crystalline structures and various categories of synthesis reactions, POFs can be subdivided into crystalline covalent‐organic frameworks (COFs),^2^ covalent triazine frameworks (CTFs),^3^ amorphous hypercross‐linked polymers (HCPs),^4^ polymers of intrinsic microporosity (PIMs),^5^ conjugated microporous polymers (CMPs),^6^ porous aromatic frameworks (PAFs),^7^ etc. I) Crystalline POFs: COFs represent an emerging generation of crystalline POFs that are synthesized through the assembly of organic building blocks into predictable, periodic, 2D or 3D frameworks.[[qv: 2e–g]] CTFs, a distinctive subclass of COFs, are constructed through the cyclotrimerization of rigid nitriles to develop triazine rings.[[qv: 3a–c]] II) Amorphous POFs: HCPs are primarily synthesized based on Friedel–Crafts chemistry, which provides fast kinetics to produce the strong linkages of neighboring aromatic rings, resulting in highly cross‐linked networks.[[qv: 4b]] HCPs demonstrate certain superiorities (e.g., inexpensive raw reagents and mild reaction conditions) but still demonstrate insufficiency in chemical diversity.[[qv: 1b]] PIMs, as a genre of amorphous microporous polymers, are assembled by a continuous chain of interclasped aromatic rings, with some contorted sites.[[qv: 5a]] The microporosity of PIMs is primarily attributed to their flexible polymer chains, instead of interconnected covalent bonds.[[qv: 5a]] CMPs have π‐conjugated permanently microporous skeletons,[[qv: 6c]] and their amorphous architecture originates from the rotational freedom of *s*‐bonds that form between the monomers.[[qv: 1b]] PAFs are an innovative member of the amorphous POFs family, which are synthesized by linking phenyl‐based monomers (through irreversible C—C coupling reactions) to generate rigid diamondoid or other structured frameworks.[[qv: 7a,b]]

POFs, with adjustable pore sizes, high surface areas, and easy postsynthetic functionalization, exhibit many remarkable characteristics: I) the diversity of organic building monomers and condensation reactions promotes the facilitation of the synthesis of POFs with specific functions and architectures; II) POFs are condensed through the linkage of robust covalent bonds, endowing them with high chemical resistance and thermostability; III) many active sites in their frameworks are easily modified, with some functional groups; IV) the periodic crystalline structures of COFs and CTFs are beneficial to improve their performance at atomic and molecular levels. These advantages position POFs as up‐and‐coming candidate materials for gas storage^8^ and separation,^9^ semiconduction,^10^ proton conduction,^11^ catalysis,^12^ energy storage,[[qv: 3d,13]] drug delivery,^14^ and other important applications.

**Figure 1:**
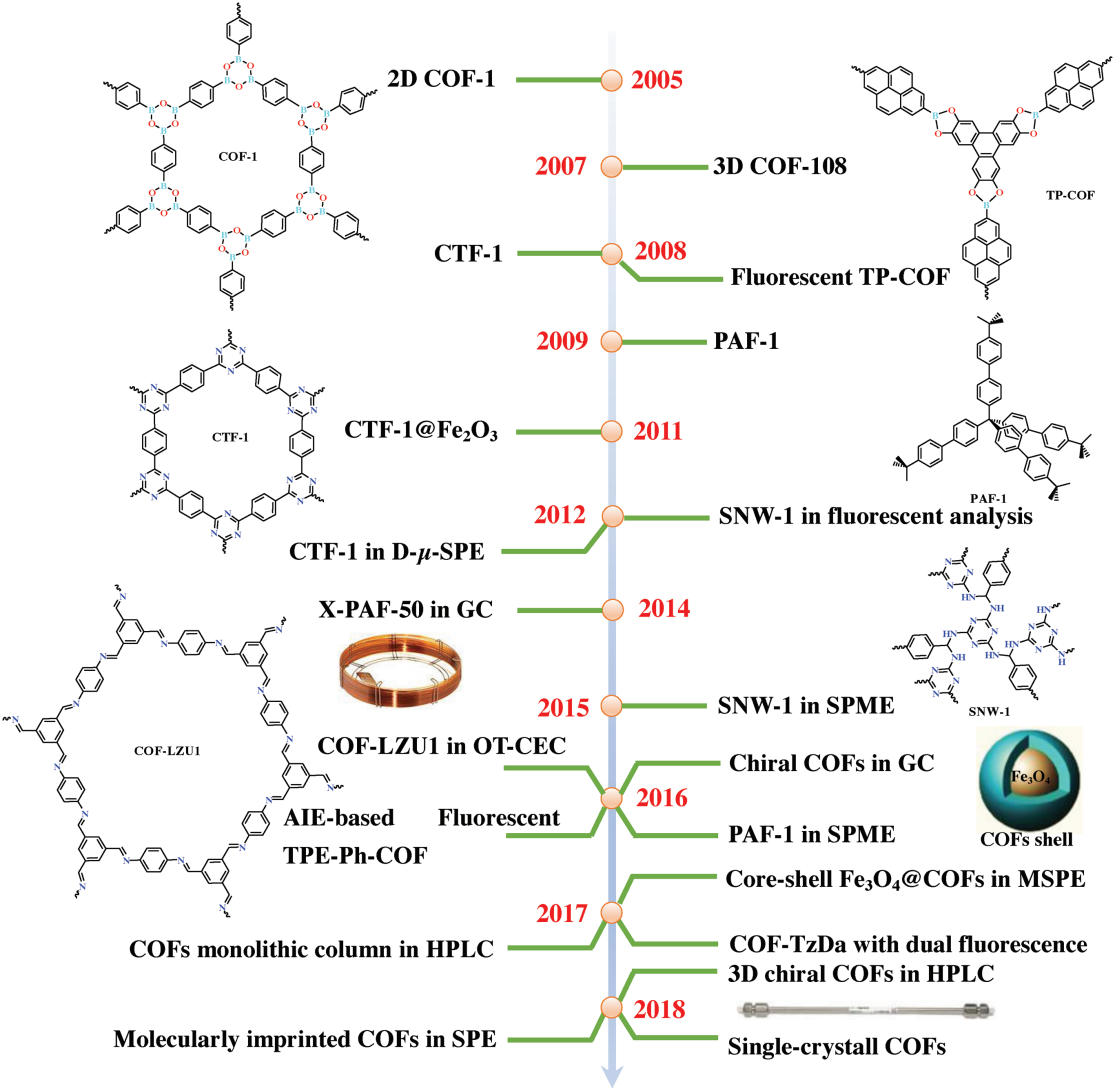
Milestones of POFs (COFs, CTFs, and PAFs) and their applications in analytical chemistry‐related fields.

Analytical chemistry is defined as the art of pretreating, separating, recognizing different constituents, and determining substances from samples, which evolved from an art to a branch of chemical science with great theoretical and practical value for diverse applied sciences and technologies.^15^ Undoubtedly, as advanced materials, POFs have also gradually triggered significant research interest in analytical chemistry, due to their fascinating characteristics. Considering the significant progress of COFs, CTFs, and PAFs in analytical chemistry, herein, we highlight and summarize recent advancements in the synthesis of these POFs and their utilizations in fluorescence detection, chromatographic separation, and sample preparation, three of the dominant research fields in analytical chemistry. I) To date, POFs have shown great potential for use in “turn‐off” or “turn‐on” fluorescence detection of nitrobenzene derivatives, small molecules, metal ions, etc. II) POFs have been increasingly utilized as the novel stationary phases in chromatographic separation systems, including gas chromatography (GC), high‐performance liquid chromatography (HPLC), and capillary electrochromatography (CEC). III) The combination of sample preparation and POFs has led to effective improvements in the capability of these techniques, such as on‐line solid phase extraction (SPE), dispersive microsolid phase extraction (D‐µ‐SPE), magnetic solid‐phase extraction (MSPE), solid‐phase microextraction (SPME), etc. **Figure** **1** shows important advancements in the synthesis of COFs, CTFs, as well as PAFs and their applications in analytical chemistry. In this review, some limitations related to the utilization of POFs as a novel porous material in analytical chemistry are also outlined, in addition to providing directions on how to surmount these issues. Furthermore, the future possibilities and potentials of COFs, CTFs, and PAFs as advanced materials in analytical chemistry are discussed.

## 2 COFs, CTFs, and PAFs

The three important family members of porous organic frameworks, COFs, CTFs, and PAFs are summarized in this section, and their creation, development, and synthetic techniques, as well as the specific features of each family member are also highlighted.

### 2.1 COFs

COFs, which are endowed with unique crystallinity, as well as porosity, represent a momentous branch of POFs that enabled atomically precise integration of organic monomers into crystalline architectures through robust covalent bonds.^2^ The pioneering synthesis of COFs, 2D COF‐1 (*S*_BET_ = 711 m^2^ g^−1^), and COF‐5 (*S*_BET_ = 1590 m^2^ g^−1^) was created by Yaghi and co‐workers[[qv: 2a]] in 2005: COF‐1 was synthesized through the self‐condensation of benzenediboronic acid (BDBA), while COF‐5 was synthesized through the cocondensation of BDBA and hexahydroxytriphenylene (HHTP). Later, the family of 2D‐COFs began to germinate, as COF‐6 (*S*_Langmuir_ = 980 m^2^ g^−1^), COF‐8 (*S*_Langmuir_ = 1400 m^2^ g^−1^), and COF‐10 (*S*_Langmuir_ = 2080 m^2^ g^−1^) were successively constructed through the cocondensations of HHTP with different boronic‐acids monomers.[[qv: 2b]] The Dichtel group^16^ reported the growth of 2D‐COF films on single‐layered graphene, with improved crystallinity and long range order. Of particular note, the first 3D‐COFs was also pioneered by Yaghi and co‐workers[[qv: 2c]] through the self‐condensation reactions of tetrahedral tetra(4‐dihydroxyborylphenyl)methane (TBPM), its silane analog (TBPS), and triangular HHTP to yield crystalline COF‐102 (*S*_BET_ = 3472 m^2^ g^−1^), COF‐103 (*S*_BET_ = 4210 m^2^ g^−1^), COF‐105, and COF‐108, respectively.

In general, COFs are subdivided into seven categories, in terms of the covalent bonds that the organic monomers formed.[[qv: 2h,i]] I) B—O: boroxine (such as COF‐1),[[qv: 2a]] boronate ester (COF‐5),[[qv: 2a]] borosilicate (COF‐202, *S*_BET_ = 2690 m^2^ g^−1^),^17^ and spiroborate (ionic‐COF, *S*_BET_ = 1259 m^2^ g^−1^)^18^; II) C=N: imine COFs[[qv: 2k,12a,19]] such as COF‐300 (*S*_BET_ = 1360 m^2^ g^−1^),[[qv: 19a]] COF‐LZU1 (*S*_BET_ = 410 m^2^ g^−1^),[[qv: 12a]] TAPB‐PDA (*S*_BET_ = 600 m^2^ g^−1^),[[qv: 19b]] 3D‐Py‐COF (*S*_BET_ = 1290 m^2^ g^−1^),[[qv: 19c]] COF‐505,[[qv: 19d]] 3D‐Por or 3D‐CuPor‐COF (*S*_BET_ = 1335–1398 m^2^ g^−1^),[[qv: 19e]] 3D‐ionic‐COFs (*S*_BET_ = 880–996 m^2^ g^−1^),[[qv: 19f]] BND‐TFB COF (*S*_BET_ = 2618 m^2^ g^−1^),[[qv: 19g]] COF‐LZU‐111 (*S*_BET_ = 2120 m^2^ g^−1^),[[qv: 2k]] BDT‐ETTA COF (*S*_BET_ = 1360 m^2^ g^−1^),[[qv: 19h]] TPB‐DMTP COF (*S*_BET_ = 1927 m^2^ g^−1^),[[qv: 19i]] and TTA‐TTB COF (*S*_BET_ = 1733 m^2^ g^−1^)[[qv: 19i]]; azine (Py‐Azine COF, *S*_BET_ = 1210 m^2^ g^−1^)^20^; hydrazone (COF‐42, *S*_BET_ = 710 m^2^ g^−1^, and COF‐43, *S*_BET_ = 620 m^2^ g^−1^),[[qv: 2d]] and squaraine (CuP‐SQ COF, *S*_BET_ = 2289 m^2^ g^−1^);^21^ III) C=N (aromatic): triazine (CTF‐1, *S*_BET_ = 791 m^2^ g^−1^),[[qv: 3a]] and phenazine (CS‐COF, *S*_BET_ = 776 m^2^ g^−1^);^22^ IV) C—N: β‐ketoenamine COFs[[qv: 13a,23]] such as DAAQ‐TFP (*S*_BET_ = 435 m^2^ g^−1^),[[qv: 13a]] DAB‐TFP (*S*_BET_ = 365 m^2^ g^−1^),[[qv: 13a]] TAPB‐TFP (*S*_BET_ = 567 m^2^ g^−1^),[[qv: 23a]] *i*PrTAPB‐TFP (*S*_BET_ = 756 m^2^ g^−1^),[[qv: 23a]] TP‐EDDA (*S*_BET_ = 523 m^2^ g^−1^),[[qv: 23b]] and TP‐BDDA (*S*_BET_ = 758 m^2^ g^−1^);[[qv: 23b]] polyimide COFs[[qv: 14a,24]] such as PI‐COF‐1‐PI‐COF‐5 (*S*_BET_ = 1027–2403 m^2^ g^−1^),[[qv: 14a,24a]] PIBN‐G,[[qv: 24b]] and PIBN;[[qv: 24b]] amide COFs^25^ such as TPB‐TP‐COF (*S*_BET_ = 655 m^2^ g^−1^),[[qv: 25a]] 4PE‐1P‐COF (*S*_BET_ = 1190 m^2^ g^−1^),[[qv: 25a]] and CCOF‐6 (*S*_BET_ = 613 m^2^ g^−1^);[[qv: 25b]] V) B=N: borazine (BLP‐2(H), *S*_BET_ = 1178 m^2^ g^−1^)^26^ or N=N (azodioxy POR‐COF, *S*_BET_ = 447 m^2^ g^−1^);^27^ VI) C=C: alkene 2DPPV COF (*S*_BET_ = 472 m^2^ g^−1^);^28^ VII) Si—O:^29^ 2D SiCOFs (M_2_[Si(C_16_H_10_O_4_)_1.5_], M represents K, Na, and Li, *S*_BET_ = 1067–1276 m^2^ g^−1^)[[qv: 29a]] and 3D SiCOF‐5 (Na_2_[Si(C_18_H_6_O_6_)], *S*_BET_ = 370 m^2^ g^−1^).[[qv: 29b]]

**Figure 2:**
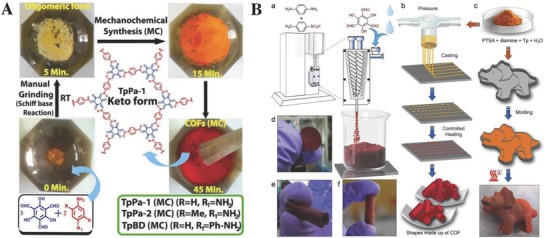
A schematic illustration of the mechanochemical synthesis of Tp‐COF (e.g., TpPa‐1) through A) grinding, B‐a) extrusion, B‐b) terracotta, or B‐c) organic terracotta techniques by Banerjee and co‐workers. Adapted with permission.^32^ Copyright 2013^32^ and 2017^32^ American Chemical Society.

From a synthetic perspective, COFs are usually prepared under the following methods: solvothermal, microwave‐assisted solvothermal, mechanochemical, and room temperature synthesis in solution.[[qv: 2g]] I) Solvothermal synthesis is a preliminary and extensively used approach for the construction of crystalline COFs, and it typically involves some harsh condensation conditions, for instance, reaction monomers are sealed in a Pyrex tube under an inert atmosphere, and the solvothermal reaction usually must be implemented at an elevated temperature for a relatively long time period of several days.[[qv: 2a]] The solvent combinations/ratios in the solvothermal condensation reactions are essential to obtain high stable and crystalline COFs. II) The microwave‐assisted solvothermal method has superiority in considerably reducing reaction times compared to the traditional solvothermal counterparts, thereby offering new feasibilities for further large scale applications.[[qv: 2g]] As demonstrated by previous reports by Cooper and co‐workers^30^ and Wei et al.,^31^ the microwave‐assisted synthesis of COF‐5 (*S*_BET_ = 2019 m^2^ g^−1^),^30^ COF‐102 (*S*_BET_ = 2926 m^2^ g^−1^)^30^ and COF‐TpPa‐1 (*S*_BET_ = 724.6 m^2^ g^−1^)^31^ was achieved in high yields with shorter reaction times of 20–60 min. III) The mechanochemical technique was a novel alternative to the traditional solvothermal synthetic routes, as this method featured the merits such as quick, easy‐to‐perform, and eco‐friendly.^32^ Banerjee and co‐workers^32^ have pioneered the introduction of mechanochemical operations (**Figure** **2**), such as grinding, extrusion, and terracotta, to fabricate a series of highly crystalline β‐ketoenamine‐based 1,3,5‐triformylphloroglucinol (Tp)‐COFs (*S*_BET_ = 538–3109 m^2^ g^−1^).[[qv: 32b]] IV) For the room temperature synthesis in solution,^33^ Zamora and co‐workers[[qv: 33a]] developed a facile and rapid room temperature method for the synthesis of an imine‐linked COF, RT‐COF‐1 (*S*_BET_ = 329 m^2^ g^−1^), through the Schiff‐based condensation of 1,3,5‐tris(4‐aminophenyl)‐benzene (TAPB) and 1,3,5‐benzenetricarbaldehyde using an acetic acid catalyst in dimethyl sulfoxide (DMSO) or methylphenol solution. This RT‐COF‐1 shows a hexagonal‐structured crystallinity and good thermal stability. Recently, the liquid–liquid interfacial methods^34^ have been developed by Banerjee et al.[[qv: 34a]] and Ma et al.[[qv: 34b]] for the room temperature construction of COFs at the interface of two solvents. The new method allows for the direct synthesis of COF thin films, such as COF‐Tp‐Bpy, Tp‐Azo, Tp‐Ttba, Tp‐Tta, and NS‐COF in a free‐standing form, and their thickness can be easily controlled. Moreover, Guan and co‐workers^35^ have reported the design and construction of some 3D ionic liquid‐containing COFs (3D‐IL‐COF‐1–3D‐IL‐COF‐3, *S*_BET_ = 517–870 m^2^ g^−1^) with 5–11‐folds interpenetrated *dia* frameworks by employing a pressure ionothermal method under ambient temperatures. These 3D‐IL‐COFs, with impressive crystallinities and selective adsorption abilities toward carbon dioxide/methane and carbon dioxide/nitrogen, had the benefits of high reaction speeds (e.g., a 3 min synthesis of 3D‐IL‐COF‐1).

### 2.2 CTFs

As an exclusive and emerging subclass of COFs, CTFs achieved scientific limelight in 2008, when the Thomas and co‐workers[[qv: 3a]] synthesized the first triazine‐based crystalline CTF‐1. Generally, CTFs are formed through the trimerization reaction of the functional nitrile groups from nitrile monomers to form triazine‐based frameworks under ionothermal conditions[[qv: 3a–c,36]] (e.g., CTF‐1 was cyclo‐trimeric synthesized from 1,4‐dicyanobenzene using a molten ZnCl_2_, which acts as both solvent and catalyst at 400 °C).[[qv: 3a]] The powder X‐ray diffraction (PXRD) pattern of CTF‐1 showed that this POF exhibits an architecture of 2D hexagonal framework like its isoelectronic COF‐1. Due to the strong Lewis acid–base effects, nitrile compounds show commendable solubility in the ionic melt of ZnCl_2_, with the formation of a clear solution in an ionothermal environment. A higher ratio of ZnCl_2_ to 1,4‐dicyanobenzene monomer (e.g., 10:1) prohibits crystallization and provides CTFs with a higher surface area. Other crystalline CTF species have been prepared by Thomas and co‐workers, for instance, CTF‐2 (*S*_BET_ = 90 m^2^ g^−1^) and *mp*‐CTF‐2 (*S*_BET_ = 2255 m^2^ g^−1^) were prepared through the trimerization of 2,6‐napthalenedinitrile[[qv: 3b]] and CTF‐0 (*S*_BET_ = 687 m^2^ g^−1^) from 1,3,5‐tricyanobenzene under ionothermal conditions similar to those of CTF‐1.[[qv: 3c]] Giambastiani and co‐workers[[qv: 36b]] have also synthesized a series of CTFs, CTF‐ph/CTF‐ph^HT^, and CTF‐py/CTF‐py^HT^ (*S*_BET_ = 1239–3040 m^2^ g^−1^) from 1,3‐dicyanobenzene (1,3‐DCB) and 2,6‐dicyanopyridine (2,6‐DCP) monomers using molten ZnCl_2_ as a catalyst. Recently, Wang et al.^37^ synthesized a series of highly microporous and nitrogen‐rich CTFs (*S*_BET_ = 802–1563 m^2^ g^−1^) through trimerization of 4,4′,4″,4‴‐(1,4‐phenylenebis[pyridine‐4,2,6‐triyl])‐tetrabenzonitrile monomer in molten ZnCl_2_ under ionothermal conditions. Although the ionothermal approach is helpful for the formation of triazine rings (C_3_N_3_) in the frameworks of CTFs,^38^ this strategy still suffers from some shortcomings (e.g., a relatively high temperature [400–700 °C] and a prolonged synthesis time [ranging from 20 h to more than 40 h, depending on the different heat treatments]).[[qv: 3a–c,36]] To shorten the reaction time, Zhang et al.[[qv: 36a]] later developed a microwave‐enhanced ionothermal approach for the preparation of CTF‐1, with a generous reduction time ranging from 40 h to 10–60 min. However, in the aforementioned cases, the ZnCl_2_ catalyst was difficult to remove from the reaction systems, which contributed to the inevitable contamination of inorganic (often metallic) residues.[[qv: 3a–c,36a]]

**Figure 3:**
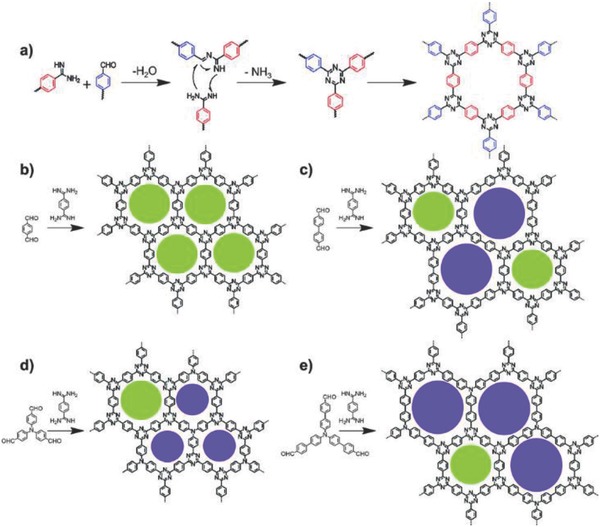
a) The reaction mechanism for CTF‐HUST synthesis by a Schiff base formation, followed by a Michael addition. Representations of the structures of b) CTF‐HUST‐1, c) CTF‐HUST‐2, d) CTF‐HUST‐3, and e) CTF‐HUST‐4. Here, the circles are filled with different colors representing the presence of two types of pores. Adapted with permission.^43^ Copyright 2017, Wiley‐VCH Verlag GmbH & Co. KGaA, Weinheim.

To address these issues, Cooper and co‐workers^39^ synthesized a series of CTFs (CTF‐P1–P6 and P1M–P6M, *S*_BET_ = 464–1152 m^2^ g^−1^, except for P1 and P1M) that originated from various aromatic nitriles using Brønsted acid (CF_3_SO_3_H) as the catalyst, under room‐temperature or microwave‐assisted conditions. Their experimental results indicated that all CTFs prepared at room temperature (CTF‐P1–P6) were found to be principally amorphous; however, some CTFs (P1M, P2M, and P4M) formed from microwave‐assisted synthesis exhibited limited crystallinity. Zhu and co‐workers^40^ synthesized a fluorescent CTF‐based membrane (triazine‐framework‐based porous membrane‐1 (TFM‐1), *S*_BET_ = 738 m^2^ g^−1^) through CF_3_SO_3_H‐catalyzed cross‐linking reactions of 4,4′‐biphenyldicarbonitrile at a lower temperature (<100 °C). With the help of functionalized triazine units in frameworks, TFM‐1 demonstrated an enhanced separation selectivity toward carbon dioxide compared to nitrogen. Later, a photoluminescent porous CTF (PCTF‐8, *S*_BET_ = 625 m^2^ g^−1^)^41^ was prepared at room temperature using the π‐conjugated tetra(4‐cyanophenyl)ethylene as a single monomer and CF_3_SO_3_H as catalyst. Recently, AlCl_3_‐catalyzed Friedel–Crafts chemistry was also used to construct CTFs from cyanuric chloride and other aromatic monomers.^42^ Friedel–Crafts chemistry method shows the significant advantages of low‐cost reagents, low synthesis temperatures, facile reaction conditions, and high yields. For instance, Dey et al.[[qv: 42b]] synthesized CTF‐TPC (*S*_BET_ = 1688 m^2^ g^−1^) and CTF‐FL (*S*_BET_ = 773 m^2^ g^−1^) using the AlCl_3_‐catalyzed Friedel–Crafts reactions between cyanuric chloride as the “node” and triptycene/fluorene as the “linkers.” The triazine frameworks of two CTFs originated from the electrophilic aromatic substitution reactions that occurred on triptycene or fluorene phenyl rings. Recently, Tan and co‐workers^43^ synthesized a series of CTFs (CTF‐Huazhong University of Science and Technology (HUSTs), *S*_BET_ = 663–807 m^2^ g^−1^) through a Schiff base formation, followed by a Michael addition reaction between aldehydes and amidines (**Figure** **3**). This novel polycondensation reaction allowed the preparation of CTFs under a relative mild condition, which efficiently avoided high temperatures or strong acids in the traditional methods. CTF‐HUSTs, with a layered architecture, possessed diverse applications in gas adsorption, photocatalysis, and sodium‐ion battery. Recently, Yu and co‐workers^44^ constructed an innovative highly crystalline CTF, *p*CTF‐1 (*S*_BET_ = 2034.1 m^2^ g^−1^) using an eco‐friendly phosphorus pentoxide‐catalyzed synthesis method. In this strategy, P_2_O_5_ catalyst was employed for the direct condensation of aromatic primary amide groups from terephthalamide monomer into *s*‐triazine rings. The *p*CTF‐1 featured high carbon dioxide (21.9 wt%, 273 K) and hydrogen (1.75 wt%, 77 K) uptake capacities at low pressure due to not only its large surface area but also its microporosity.

### 2.3 PAFs

**Figure 4:**
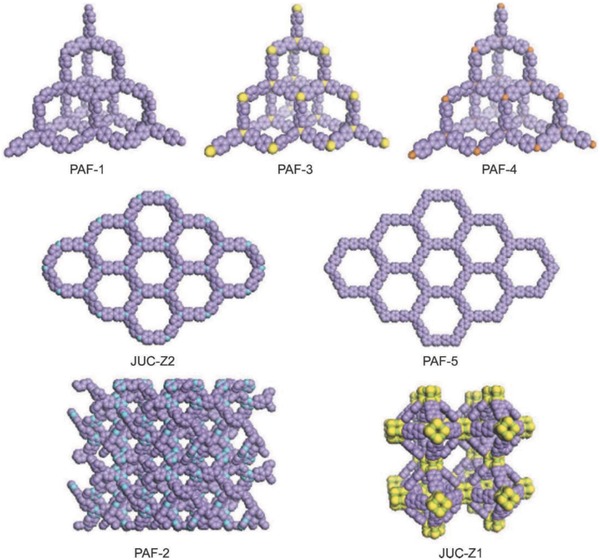
Structure models of some synthesized PAFs (C, purple; N, blue; Si, yellow, O, green, Ge, brown). Adapted with permission.[[qv: 7b]] Copyright 2013, The Royal Society of Chemistry.

In 2009, PAFs began their scientific journey with the appearance of PAF‐1,[[qv: 7a]] with excellent performances, including a record surface area (*S*_BET_ = 5640 m^2^ g^−1^), good hydrogen/carbon dioxide storage capacity, and high physicochemical stability. Inspired by the thought of the stable tetrahedral structure of diamond, Zhu and co‐workers designed PAF‐1 by breaking the C—C covalent bonds of diamond and interpolating the phenyl rings of tetrakis(4‐bromophenyl)methane monomer (structure models[[qv: 7b]] are shown in **Figure** **4**). The sufficient exposure of phenyl rings in this framework might be helpful to improve the internal surface area of PAF‐1.[[qv: 7a]] The successful design and construction of PAF‐1 encouraged the further preparation of PAF‐3, PAF‐4, and PAF‐5 through Yamamoto coupling reactions from monomers of tetrakis(4‐bromophenyl)silane, tetrakis‐(4‐bromophenyl)germane, and 1,3,5‐tris(4‐bromophenyl)benzene, respectively.[[qv: 8a,45]] These newly synthesized PAFs have showed large surfaces areas (PAF‐3, *S*_BET_ = 2932 m^2^ g^−1^; PAF‐4, 2246 m^2^ g^−1^; PAF‐5, 1503 m^2^ g^−1^) and good adsorption selectivity for both gases (PAF‐3, CO_2_/N_2_ and PAF‐4, CH_4_/N_2_)[[qv: 8a]] and organic pollutants (methanol, benzene, and toluene).^45^ Then, Zhu and co‐workers synthesized a series of PAF‐JUC‐Zs (Figure 4) based on Yamamoto's coupling reaction:^46^ JUC‐Z1[[qv: 46a]] with zeolitic LTA topology comprises *p*‐iodio‐octaphenylsilsesquioxane building blocks (*S*_BET_ = 283 m^2^ g^−1^), JUC‐Z2[[qv: 46b]] with an *hcb* topology via para‐tribromotribenzylaniline monomer (*S*_BET_ = 2081 m^2^ g^−1^), JUC‐Z4 (*S*_BET_ = 793 m^2^ g^−1^) and JUC‐Z5 (*S*_BET_ = 648 m^2^ g^−1^),[[qv: 46c]] JUC‐Z7–JUC‐Z10 (*S*_BET_ = 2885–4889 m^2^ g^−1^),[[qv: 46d]] JUC‐Z13–JUC‐Z19 (*S*_BET_ = 371–3137 m^2^ g^−1^),[[qv: 46e]] etc.

Furthermore, other coupling reactions were also used to synthesize diverse PAFs, for instance, PAF‐11 (*S*_BET_ = 704 m^2^ g^−1^)^47^ was prepared through the Suzuki–Miyaura cross‐coupling reaction between a tetrahedral unit (tetrakis‐[4‐bromophenyl]methane) and a linear linker (4,4′‐biphenyldiboronic acid). Goesten et al.^48^ also synthesized PAFs using a similar Suzuki–Miyaura coupling reaction of BDBA and 1,3,5‐tris(4‐bromophenyl)benzene or tris(4‐bromophenyl)‐amine. Zhu and co‐workers^49^ constructed a PAF‐18‐OH (*S*_BET_ = 1121 m^2^ g^−1^) and its lithium‐modified derivative (PAF‐18‐Oli, *S*_BET_ = 981 m^2^ g^−1^) through a Sonogashira–Hagihara cross‐coupling reaction. PAF‐18‐OLi shows an increased adsorption capacity of H_2_ and CO_2_ through the interactions between the lithium derived frameworks and gas molecules. Later, Zhu and co‐workers^50^ also reported the preparations of PAF‐26‐COOH, PAF‐26‐COOM (M = Li, Na, K, and Mg , *S*_BET_ = 430–717 m^2^ g^−1^), and PAF‐33–PAF‐37 (*S*_BET_ = 325–953 m^2^ g^−1^), based on Sonogashira–Hagihara cross‐coupling reactions. Additionally, PAF‐32s (*S*_BET_ = 1230–1679 m^2^ g^−1^)^51^ from tetrahedral monomers have been synthesized by the same group, based on the FeCl_3_‐catalyzed Friedel–Crafts chemistry. Zhu et al.^52^ synthesized porphyrin‐based PAF‐40s (*S*_BET_ = 601–824 m^2^ g^−1^)[[qv: 52a]] and PAF‐41–PAF‐44 (*S*_BET_ = 515–1119 m^2^ g^−1^)[[qv: 52b]] through an AlCl_3_‐catalyzed aromatic phenyl ring coupling reaction. The PAF‐40s possess a higher selectivity in the adsorption of C_2_‐ and C_3_‐hydrocarbons rather than CH_4_. The same group^53^ also prepared the fullerene‐based PAFs (PAF‐60, PAF‐61, and PAF‐62, *S*_BET_ = 701–1094 m^2^ g^−1^) using C_60_ as a novel spherical building block, which is fascinating from an architectural point of view. Remarkably, a series of molecularly imprinted porous aromatic frameworks (MIPAFs, *S*_BET_ = ≈150–781 m^2^ g^−1^) have been synthesized by Zhu and co‐workers^54^ to create porous artificial enzymes that possess excellent kinetics for guest molecules. These MIPAFs can perform a wide range of sequential processes, such as substrate hydrolysis and product transport, due to their large surface areas and abundant accessible sites.

## 3 POFs as Advanced Materials in Analytical Chemistry

New discoveries in material science always provide innovative and powerful tools for analytical chemistry. In recent years, there has been the rapid growth of POFs as promising materials for the fluorescence detection of diverse analytes, novel stationary phases for chromatography, and new absorbents for sample preparation methods. The recent developments of COFs, CTFs, and PAFs in analytical chemistry are reviewed here, and the applications of these porous materials in fluorescence detection and sample preparation techniques are summarized in **Tables** **1** and **2**, respectively. While some POFs, such as Py‐azine COF, schiff base network‐1 (SNW‐1), and, CTF‐1, can be directly used in analytical chemistry. However, most POFs must be engineered or modified to meet the specific requirements for diverse analytical applications. For instance, TfpBDH or PI‐COF must be desquamated to thin layered nanosheets for fluorescence detection. The hybrid composites of POFs@silica gel, such as CTF‐1@SiO_2_, COF‐TpBd@SiO_2_, and BtaMth‐COF@SiO_2_, were constructed for HPLC separation of various analytes of interest. Magnetic functionalized POFs with core–shell structures (e.g., Fe_3_O_4_@COF‐TpBd, Fe_3_O_4_@COF‐LZU1, Fe_3_O_4_@TpPa‐1, and Fe_3_O_4_@TAPB‐TPA) have been synthesized as novel adsorbents for MSPE. Other POFs, such as COF‐TpBd, COF‐LZU1, and PAF‐1‐NH_2_/IL, have been covalently bonded with the accessory reagents onto the capillary or fiber substrate for use as new stationary phases for GC, CEC, or fiber coatings for SPME. COFs, CTFs, and PAFs, together with their composites, exhibit tremendous application potential in analytical chemistry.

**Table 1:** Analytical applications of fluorescent POFs

POFs	Monomersa)	*S*_BET_ [m^2^ g^−1^]	Pore sizes [nm]	Analytes with high selectivityb)	Limits of detection [LODs]	Ref.
				Nitrobenzene derivatives		
Py‐azine COF	TFPPy, hydrazine	1210	1.76	TNP >> DNP > DNT, NP, NT	70 ppm	20
SNW‐1	Melamine, TPA	476	1.0	TNP >> Tetryl, TNT > DNT >> NT > NB	11.5 ppb	[[qv: 56a]]
TRIPTA	TAPT, Tp	609	1.68	TNP > DNP > DNT > NP	5.2 × 10^−8^ m	[[qv: 56b]]
COF TAPB‐TFPB	TAPB, TFPB	229	40	TNP >> DNT > *p*DNB > *m*DNB	13 ppm	[[qv: 23a]]
TfpBDH‐CONsc)	Tfp, BDH	270	–	TNP >> TNT > DNP > NP > DNT	5.4 × 10^−5^ m	[[qv: 64c]]
PI‐CONsd)	TAPP, PTCA	894	2.8	TNP >> DNP > PPD, NP > NT > DNT, NB, TNT	0.25 × 10^−6^ m	65
3D‐Py‐COF	TAPM, TFPPy	1290	0.59	TNP	20 ppm	[[qv: 19c]]
ACOF‐1	hydrazine hydrate, TFB	–	–	TNR > TNP > TNT > DNT	–	125
COP‐401 and COP‐301	DB, TBT, TBB	–	–	TNP >> TNT > DNT > *m*DNB > NB	0.68 ppm	126
PCTF‐8	Tetra(4‐cyanophenyl)ethylene	625	0.5–1.48	TNP >> NT > NB > DNP > 2,6‐DNT > 2,4‐DNT	<1.0 × 10^−5^ m	41
COF TFPC‐NDA	TFPC, NDA	294	0.75–2.1	TNP	68 ppb	127
PAF‐14	TBPGe	1288	microporous	NB, 2,4‐DNT, TNT	–	128
PAF‐15	TBPGe, HHTP	747	–	NB, 2,4‐DNT, TNT	–	129
PP_C_‐PPy_S_‐PAFs	BDBA, TBB, TBrPy	388–415	–	TNP > TNT > DNT > Cl‐NB > NT	<75 ppm	130
				Small molecules		
TAT‐COF‐2	2‐CHO‐TAT, 2‐NH_2_‐TAT	1166.1	1.48	Arene vapors	–	66
NUS‐20	TPE‐1, TBPM	900	1.23	Arene vapors	–	67
TPE‐Ph COF	TPEBA, THB	962	1.3–2.6	Ammonia	<1 ppm	61
COF‐TzDa	Tz, Da	709	3.6	Water	0.006%	68
COF‐JLU4	DMTPH, Tp	923	2.2	H^+^ in water	pH 0.9–13.0	69
				Metal ions		
COF‐LZU8	Thioether Monomer, TFB	454	1.3	Hg^2+^	25 ppb	[[qv: 73a]]
TAPB‐BMTTPA‐COF	TAPB, BMTTPA	1934	3.2	Hg^2+^	0.01 ppm	[[qv: 73b]]
COF‐S‐SH	Dva, TAPB, 1,2‐ethanedithiol	546	–	Hg^2+^	0.1 ppb	74
TPB‐DMTP‐COF‐SH	DMTA, BPTA, TAPB, 1,2‐bis(2azidoethyl)disulfane	291	2.14	Hg^2+^	1.5 µg L^−1^	131
CTFQD	1,4‐dicyanobenzene	–	–	Hg^2+^	0.23 × 10^−6^ m	132
PAF‐1‐SH	2,2′‐bipyridyl, TBPM, NaHS	3274	0.3	Hg^2+^	0.04 ppb	133
NS‐COF	Tp, DHBD	–	–	UO_2_^2+^	0.2 ppm	[[qv: 34b]]
PPN‐6‐PAN	2,2′‐bipyridyl, TBPM, acrylonitrile	19.5	–	UO_2_^2+^	≈80 ppb	134
COF‐JLU3	TBHFB, hydrazine hydrate	570	–	Cu^2+^	0.31 × 10^−6^ m	76
PI‐COF‐201, PI‐COF‐202	Melamine, PMDA, NTDA	3.9–9.2	1.34–1.41	Fe^3+^	0.13 × 10^−6^ m	77

^a)^ BDBA: benzene‐1,4‐diboronic acid; BDH: pyromellitic‐*N*,*N*′‐bisaminoimide; BMTTPA: 2,5‐Bis(methylthio)terephthalaldehyde; BPTA: 2,5‐bis(prop‐2‐in‐1‐yloxy)terephtaldehyde; 2‐CHO‐TAT: 2,7,12‐triformyl‐5,10,15‐triethyltriindole; Da: 2,5‐dihydroxyterephthalaldehyde; DB: 1,4‐Dibromobenzene; DHBD: dihydroxybenzidine; DMTA: 2,5‐dimethoxyterephtaldehyde; DMTPH: 2,5‐dimethoxyterephthalohydrazide; Dva: 2,5‐divinylterephthalaldehyde; HHTP: 2,3,6,7,10,11‐hexahydroxytriphenylene; NDA: naphthalenediamine; 2‐NH_2_‐TAT: 2,7,12‐ triamino‐5,10,15‐triethyltriindole; NTDA: naphthalenetetracarboxylic dianhydride; PMDA: pyromellitic dianhydride; PTCA: perylenetetracarboxylic dianhydride; TAPB: 1,3,5‐tris(4′‐aminophenyl)benzene; TAPM: tetra(*p*‐aminophenyl)methane; TAPP: tetra(4‐aminophenyl) porphyrin; TAPT: 1,3,5‐tris‐(4‐aminophenyl)triazine; TBB: 1,3,5‐tris(4‐bromophenyl)benzene; TBHFB: 1,3,5‐tris(3′‐*tert*‐butyl‐4′‐hydroxy‐5′‐formylphenyl) benzene; TBPGe: tetra(4‐dihydroxyborylphenyl)germanium; TBPM: tetrakis(4‐bromophenyl)methane; TBrPy: 1,3,6,8‐tetrabromopyrene; TBT: 2,4,6‐Tris‐(4‐bromophenyl)‐[1,3,5]triazine; TFB: 1,3,5‐Triformylbenzene; TFPB: 1,3,5‐tris(4′‐formylphenyl)benzene; TFPC: tri(4‐formyl phenoxy)cyanurate; TFPPy: 1,3,6,8‐tetrakis(4‐formylphenyl)pyrene; Tfp: 1,3,5‐tris(4‐formylphenyl)benzene; THB: 1,2,4,5‐tetrahydroxybenzene; Tp: 1,3,5‐triformylphloroglucinol; TPA: terephthalaldehyde; TPE‐1: 1,2‐diphenyl‐1,2‐bis(4‐(4,4,5,5‐tetramethyl‐1,3,2‐dioxaborolan‐2‐yl)phenyl)ethane; TPEBA: tetraphenylethene‐cored boronic acids; Tz: 4,4′,4″‐(1,3,5‐triazine‐2,4,6‐triyl)trianiline

^b)^ Cl‐NB: 4‐chloro‐nitrobenzene; DNP: dinitrophenol; DNT: 2,4‐dinitrotoluene; *m*‐DNB: m‐dinitrobenzene; NB: nitrobenzene; NP: nitrophenol; NT: nitrotoluene; *p*‐DNB: *p*‐dinitrobenzene; Tetryl: 2,4,6‐trinitrophenylmethylnitramine; TNP: 2,4,6‐trinitrophenol (picric acid); TNR: 2,4,6‐trinitroresorcinol; TNT: 2,4,6‐trinitrotoluene

^c)^ The thickness of TfpBDH‐CONs are 1.5–5.1 nm

^d)^ *S*_BET_ and pore sizes here are the values of PI‐COFs and the thickness of PI‐CONs are ≈1 nm.

**Table 2:** Application of POFs for sample pretreatment techniques

Sample pretreatment methods	POFs	Monomersa)	*S*_BET_ [m^2^ g^−1^]	Pore sizes [nm]	Analytesb)	Analytical instrumentsc)	LODs or maximum sorption capacity	Ref.
SPE	CTF‐1	1,4‐dicyanobenzene	789	1.23	Nitroimidazoles	HPLC‐UV	0.11–0.13 µg L^−1^	91
SPE	CTpBd COFs	cTp, benzidine	114	≈2	Metal ions	ICP‐MS	2.1–21.6 ng L^−1^	92
µSPE	HL‐COP	BTCH, TPA	87	–	Sudan dyes	HPLC‐UV	0.03–0.15 µg L^−1^	93
SPE	COF TpAzo	Tp, 4,4′‐azodianiline	636	2.57	Benzoylurea insecticides	HPLC‐UV	0.1–0.2 ng mL^−1^	94
SPE	MICOFs	TPBA, Tp, fenvalerate	432	6.5	Cyano pyrethroids	HPLC‐DAD	0.011–0.018 ng g^−1^	95
SPE	PAF‐6	Cyanuric chloride, piperazine	–	–	Bisphenol A	HPLC‐FLD	0.1 ng mL^−1^	135
D‐µ‐SPE	CTF‐1	1,4‐dicyanobenzene	782.4	1.2	Aromatic compounds	HPLC‐UV	–	97
D‐µ‐SPE	CTF‐1	1,4‐dicyanobenzene	490	1.3	Cd^2+^	FAAS	29.26 mg g^−1^ d)	136
D‐µ‐SPE	COF‐HBI	Trimesoyl chloride, PDA, HBI	66.5	6.52	U(VI) ion	ICP‐AES	81 mg g^−1^ d)	98
D‐µ‐SPE	MP‐COF	HCCP, PDA	27.2	1.0–2.1	U(VI) ion	ICP‐AES	57 mg g^−1^ d)	137
D‐µ‐SPE	MIPAF‐11s	TFPB, *p*‐divinylbenzene	95–524	–	U(VI) ion	ICP	37.28 mg g^−1^ d)	101
D‐µ‐SPE	PAF‐10s	Pb^2+^ imprinted complex, divinylbenzene, TBSF	87–613	–	Pb^2+^	ICP	90.36 mg g^−1^ d)	138
D‐µ‐SPE	TpPa‐1	Tp, Pa‐1	–	–	*N*‐linked glycopeptides	MALDI‐TOF‐MS and LC‐MS/MS	178 mg g^−1^ d)	102
D‐µ‐SPE	TpPa‐2‐Ti^4+^	Tp, Pa‐2, Ti^4+^	470	2.8	Phosphopeptides	LC‐MS/MS	4 fmol	103
D‐µ‐SPE	TpBd‐Me_2_ COF	Tp, *o*‐tolidine	468	≈2	Okadaic acid	SPATT devices	61 mg g^−1^ d)	139
D‐µ‐SPE	3D‐COOH‐COF	TFPM, DHBD, SA	540	0.68	Nd^3+^	UV–vis	0.71 mmol g^−1^	140
MSPE	Fe_3_O_4_@TpBd	Tp, benzidine	272.6	1.7	Bisphenols	UV–vis	160.6 and 236.7 mg g^−1^ d)	[[qv: 110b]]
MSPE	Fe_3_O_4_@TpBd	Tp, benzidine	114.55	5.34	PAHs	HPLC‐DAD	0.83–11.7 ng L^−1^	141
MSPE	Fe_3_O_4_@PDA@TbBd	Tp, benzidine	146.47	2.6	PAEs	GC‐MS	2.5–10 ng L^−1^	142
MSPE	Fe_3_O_4_@COF‐1	BDBA	–	–	Paclitaxel, PAHs	HPLC‐UV	0.02 ng mL^−1^	[[qv: 110c]]
MSPE	Fe_3_O_4_@PEI@COF‐LZU1	TFB, PDA	–	–	PAHs	HPLC‐UV/FLD	0.2–20 ng L^−1^	[[qv: 110d]]
MSPE	Fe_3_O_4_@COF‐LZU1	TFB, PDA	872	1.1–1.3	Iodine	UV–vis	797 mg g^−1^ d)	111
MSPE	NH_2_‐Fe_3_O_4_@TpPa‐1	Tp, Pa‐1	247.8	0.4–2.0	PAHs	HPLC‐FLD	0.24–1.01 ng L^−1^	112
MSPE	Fe_3_O_4_@TAPB‐TPA	TAPB, TPA	181.36	≈3.6	Bisphenols	HPLC‐MS	1.0–78.1 ng L^−1^	113
MSPE	Fe_3_O_4_@COF‐Apt	Trimesoyl chloride, PDA, aptamere)	42–185	10–50	OH‐PCBs	HPLC‐MS	2.1 pg mL^−1^	143
MSPE	Fe_3_O_4_@TAPB‐TPA	TAPB, TPA	178.87	≈3.6	Peptides	HPLC‐Q‐TOF/MS	5 ng µL^−1^	144
MSPE	Fe_3_O_4_@TbBd	Tb, benzidine	196.21	≈2.8	Peptides	HPLC‐UV and HPLC‐Q‐TOF/MS	0.5 ng µL^−1^	114
MSPE	Fe_3_O_4_@TpPa‐1	Tp, Pa‐1	186	3.6	*N*‐glycopeptides	MALDI‐TOF‐MS	28 fmol	[[qv: 115a]]
MSPE	Magnetic graphene @COF‐5	HHTP, PBA	201	1.1–1.8	*N*‐glycopeptides	MALDI‐TOF‐MS	0.5 fmol µL^−1^	[[qv: 115b]]
MSPE	Magnetic graphene@ TpPa‐1	Tp, Pa‐1	–	–	Trypsin	MALDI‐TOF‐MS	268 mg g^−1^ d)	[[qv: 115c]]
MSPE	Fe_2_O_3_/CTF‐1	1,4‐dicyanobenzene	1149	2.0	Methyl orange	UV–vis	291 mg g^−1^ d)	116
MSPE	Ni/CTF‐1	1,4‐dicyanobenzene	239	–	PAEs	GC‐FID	0.024–0.085 µg g^−1^	117
MSPE	Fe_3_O_4_@SiO_2_‐PTMS@CTF	Cyanuric chloride, biphenyl	–	1.3	Parabens	HPLC‐UV	0.02 µg L^−1^	145
MSPE	Fe_3_O_4_@SiO_2_@PAF‐6	Cyanuric chloride, piperazine	120.2	2–5	Phenols, PAHs, and nitroaromatics	HPLC‐UV/FLD	0.08–5.02 ng mL^−1^	146
MSPE	MOP‐2	BD, *m*‐trihydroxybenzene	327	Mesopore	Methylene blue >> methyl orange	UV–vis	1153 mg g^−1^ d)	[[qv: 118a]]
MSPE	MOP‐SH	TAPB, 1,4‐benzenedithiol	270	11	Hg(II)	ICP‐OES	703 mg g^−1^ d)	[[qv: 118b]]
MSPE	M‐PPOP	Pyrrole, TPA	310	–	PUHs	HPLC‐DAD	0.1–0.2 µg L^−1^	147
SPME	SNW‐1	Melamine, TPA	231	≈1.4	PAHs, VFAs	GC‐MS	0.014–0.026 µg L^−1^	[[qv: 123a]]
SPME	SNW‐1	Melamine, TPA	668	≈2.8	Phenols	GC‐MS	0.06–0.2 ng g^−1^	[[qv: 123c]]
SPME	Hydrazine COF	BTCA, TPDH	722	≈1.2	Pyrethroids	GC‐ECD	0.11–0.23 µg kg^−1^	[[qv: 123g]]
SPME	Hydrazine COF	BTCH, HPA	699	≈11	OCPs	GC‐ECD	0.3–2.3 pg kg^−1^	[[qv: 123h]]
SPME	COF‐SCU1	Trimesoyl chloride, PDA	65.3	27.2	Benzene homologues	GC‐MS	0.03–0.15 ng L^−1^	[[qv: 123e]]
SPME	PAF‐1‐NH_2_/ Ionic liquid	TBPM	–	–	OCPs	GC‐ECD	0.11–0.29 µg L^−1^	[[qv: 123i]]
SPME	PAF‐48/gel	TPB	1308	0.54–1.17	Styrene, benzene homologues	GC‐FID	0.003–0.06 ng g^−1^	[[qv: 123f]]
SPME	PAF	BPDBA, melamine	–	–	Antioxidants, preservatives	GC‐FID	0.12–0.30 µg L^−1^	[[qv: 123j]]
SPME	PAF‐6	Cyanuric chloride, piperazine	159	2.06	PAHs, PAEs, and *n*‐alkanes	GC‐MS	0.8–4.2 ng L^−1^	[[qv: 123b]]
SPME	POP‐1‐ POP‐3	Phloroglucinol, TPA	193–482	1.05–8.67	PAHs, BTEX	GC‐MS	0.10–0.29 ng L^−1^	124
SPME	JUC‐Z2	TBPA	1581	0.73–1.18	Aromatic amines	GC‐MS/MS	0.010–0.012 ng L^−1^	148
SBSEf)	PDMS/CTF‐1	1,4‐dicyanobenzene	789	1.23	Phenols	HPLC‐DAD	0.08–0.3 µg L^−1^	[[qv: 123d]]

^a)^ BPDBA: 4,4′‐biphenyldiboronic acid; BTCA: 1,3,5‐benzenetricarboxaldehyde; BTCH: 1,3,5‐benzenetricarbohydrazide; cTp: 1,3,5‐triformylphloroglucinol modified by ‐COOH groups; HBI: 2‐(2,4‐dihydroxyphenyl)‐benzimidazole; HCCP: hexachlorocyclotriphosphazene; HPA: 4‐hydroxyisophthalaldehyde; Pa‐1: *p*‐phenylenediamine; Pa‐2: 2,5‐dimethyl‐1,4‐benzenediamine; PBA: phenylboronic acid; PDA: *p*‐phenylenediamine; SA: succinic anhydride; Tb: 1,3,5‐triformylbenzene; TBSF: 2,2′,7,7′‐tetrabromo‐9,9′‐spirobifluorene; TFB: 1,3,5‐triformylbenzene; TPB: 1,3,5‐triphenylbenzene; TPDH: Terephthalic dihydrazide; TBPA: Tris(4‐bromophenyl)amine

^b)^ BTEX: benzene, toluene, ethylbenzene and *m*, *o*, *p*‐xylene; OCPs: organochlorine pesticides; OH‐PCBs: hydroxylated polychlorinated biphenyls; PAEs: phthalic acid esters; PAHs: polycyclic aromatic hydrocarbons; PUHs: phenylurea herbicides; VACs: volatile aromatic compounds; VFAs: volatile fatty acids

^c)^ FAAS: flame atomic absorption spectrophotometer; HPLC‐DAD: high‐performance liquid chromatography with a diode‐array detector; HPLC‐FLD: HPLC with a fluorescence detector; HPLC‐Q‐TOF/MS: HPLC‐quadrupole‐time‐of‐flight mass spectrometry; HPLC‐UV: HPLC with an ultraviolet detector; GC‐ECD: gas chromatography with an electron capture detector; GC‐FID: GC with a flame ionization detector; GC‐MS: GC with a mass spectrometry detector; GC‐MS/MS: GC‐tandem mass spectrometery; ICP‐AES: Inductively coupled plasma atomic emission spectroscopy; ICP‐MS: inductively coupled plasma mass spectrometry detection; ICP‐OES: inductively coupled plasma optical emission spectrometer; MALDI‐TOF‐MS: matrix‐assisted laser desorption ionization‐time of flight‐mass spectrometry; UV‐vis: UV‐visible spectrophotometer; SPATT, solid‐phase adsorption toxin tracking

^d)^ The maximum sorption capacity of POFs to analytes

^e)^ The sequence of aptamer: 5′ NH2‐AGC‐AGC‐ACA‐GAGGTC‐AGA‐TGC‐ACT‐CGG‐ACC‐CCA‐TTC‐TCC‐TTC‐CAT‐CCC‐TCA‐TCCGTC‐CAC‐CCT‐ATG‐CGT‐GCT‐ACC‐GTG‐AA

^f)^ SBSE: stir bar sorptive extraction.

### 3.1 Fluorescence Spectrometric Characteristics and Analytical Applications

There has been considerable research on the fluorescence spectrometric characteristics of POFs, based on the fluorescence quenching (turn‐off)[[qv: 19c]] or enhancement (turn‐on)^55^ by guest molecules or ions. These successful applications have demonstrated that fluorescent POFs could be promising candidates for the fluorescence detection of diverse analytes. This section describes in detail the synthesis of fluorescent POFs (particularly COFs), the potential of fluorescence analysis, and the relevant investigation progress of fluorescent POFs.

#### 3.1.1 The Synthesis of Fluorescent POFs

POFs with fluorescence were primarily synthesized through different condensation reactions of aromatic monomers, such as pyrene,^10^, ^20^ triphenylene,[[qv: 2c,10a]] triazine,^39^, ^56^ *s*‐tetrazine,^57^ fluoranthene,^58^ porphyrin,^59^ phthalocyanine,^55^, ^60^ and so on. The high electro‐delocalization in the large π‐conjugated systems of fluorescent POFs promises a high probability, which allows electrons to transition from the ground state (*s*_0_) to the first excited singlet state (*s*_1_), resulting in a higher π* to π radiation relaxation probability than the electrons recovered from *s*_1_ to *s*_0_ to generate fluorescence.[[qv: 60c]] Jiang and co‐workers pioneered to construct a number of fluorescent POFs, such as TP‐COF (*S*_BET_ = 868 m^2^ g^−1^),[[qv: 10a]] PPy‐COF (*S*_BET_ = 932 m^2^ g^−1^),[[qv: 10b]] Py‐Azine‐COF (*S*_BET_ = 1210 m^2^ g^−1^),^20^ CuP‐SQ‐COF,^21^ MP‐COFs (M = H_2_, Zn, and Cu, *S*_BET_ = 1713–1894 m^2^ g^−1^),[[qv: 59a]] porphyrin‐COFs (*S*_BET_ = 1094 m^2^ g^−1^),[[qv: 59b]] NiPc‐COF (*S*_BET_ = 624 m^2^ g^−1^),[[qv: 60a]] 2D‐NiPc‐BTDA‐COF (*S*_BET_ = 887 m^2^ g^−1^),^55^ TPE‐Ph (*S*_BET_ = 962 m^2^ g^−1^),^61^ etc. TP‐COF[[qv: 10a]] and PPy‐COF,[[qv: 10b]] two of the earliest fluorescent POFs, were synthesized through the cocondensation or self‐condensation of π‐conjugated pyrene derivatives. The belt‐shaped TP‐COF showed good semiconducting features and intense blue light emission ability,[[qv: 10a]] while PPy‐COF could generate prominent photocurrent under the irradiation of light and emit a blue‐luminescence under exposure to visible lights.

Porphyrins,^59^ as 18‐electron planar macrocyclic monomers, are also appropriate for the synthesis of fluorescent POFs. For instance, 2D metalloporphyrin COFs with different central H_2_ or metals (Zn or Cu) have been synthesized using a flash‐photolysis time‐resolved microwave conductivity method.[[qv: 59a]] These MP‐COFs, ZnP‐COF in particular, have a remarkable photo‐current generated under the irradiation of visible or near infrared lights. Imine‐linked porphyrin COFs (MP‐DHPh, *S*_BET_ = 916–1054 m^2^ g^−1^) with adjustable hydrogen‐bonds sites were also synthesized by Jiang and co‐workers.[[qv: 59b]] The interactions of intralayer hydrogen‐bonds ensured the formation of 2D planar structured COFs with an enhanced π‐conjugated system, as well as an increased light‐harvesting capability. In addition, phthalocyanines, with extensive absorption spectrums, can be utilized as fascinating building monomers for the preparation of fluorescent POFs. Phthalocyanine‐based COFs (NiPc‐COF)[[qv: 60a]] have an increased capability of harvesting lights ranging from deep‐red visible to near‐infrared regions. Meanwhile, phthalocyanine‐based ZnPc‐COFs[[qv: 60b,62]] or Co/CuPc‐COFs^62^ have also been designed and prepared by Jiang and co‐workers^62^ and Dichtel and co‐workers,[[qv: 60b]] with *S*_BET_ ranging from 420 to 1360 m^2^ g^−1^.

#### 3.1.2 The Turn‐Off or Turn‐On Mode in Fluorescence Detection

The mechanism of fluorescence detection is primarily implemented through a pattern of fluorescence quenching (“turn‐off”) of the electron‐withdrawing analysts (as guest acceptors) toward the fluorescent POFs (as donors). These guest acceptors, such as nitrobenzene derivatives and metal ions, are supposed to have appropriate acceptor energy levels (e.g., the lowest unoccupied molecular orbital [LUMO] of the acceptor molecules[[qv: 57a]] or unfilled d‐orbitals of high electrovalent metal ions).^63^ This dynamic quenching was theoretically possible because the emission bands of the POFs partially overlapped with the absorption bands of the guest acceptors.[[qv: 60c]] In this “turn‐off” mode, the interactions between the guest acceptors and fluorescent POFs, such as coordination^63^ and hydrogen bands,^20^ possibly destructed the conjugation architectures and/or reduced the π‐electron delocalization systems, which resulted in the fluorescence quenching.[[qv: 60c]]

**Figure 5:**
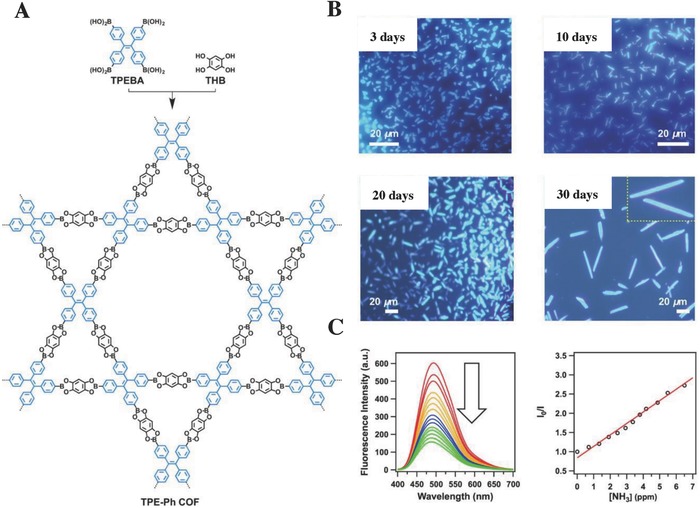
A) A schematic representation for the preparation of a “turn‐on” TPE‐Ph‐COF using an AIE‐active TPE unit. B) The fluorescence microscopy images of the TPE‐Ph‐COF were synthesized at reaction times of 3‐, 10‐, 20‐, and 30‐days. C) The fluorescence spectrum changes of TPE‐Ph‐COF with the addition of NH_3_ and the Stern–Volmer plot of the fluorescence quenching by NH_3_. Adapted with permission.^61^ Copyright 2016, American Chemical Society.

The aggregation states of the fluorescent materials play an important role in their fluorescence characteristics in two manners: “aggregation‐caused quenching” (ACQ) or “aggregation‐induced emission” (AIE).^64^ As opposed to the “turn‐off” ACQ‐fluorescent POFs, the “turn‐on” AIE‐POFs are nearly nonemissive in the molecular state but emit strongly in the aggregated state, which provides higher sensitivity and accuracy.^64^ Although turn‐on fluorescence is less common than turn‐off quenching, it reflects the future development direction of fluorescent analytical methods.[[qv: 64b,c]] The “turn‐on” AIE‐POFs can be operated in the following manner: an AIE‐POF is conjugated with a targeting ligand that can selectively bind to the analytes of interest, which restrict the molecular motions of the POFs to “turn on” fluorescence.[[qv: 64b]] In 2016, Jiang and co‐workers^61^ designed a “turn‐on” AIE‐POF, TPE‐Ph‐COF (*S*_BET_ = 962 m^2^ g^−1^), by introducing AIE‐active tetraphenylethene (TPE)‐modified boronic acids (TPEBA) to the solvothermal condensation, with 1,2,4,5‐tetrahydroxybenzene (THB). As shown in the schematic representation (**Figure** **5**A), the four phenyl groups of the TPE vertices connect to different linkers, which considerably slow down the rotation freedom of the phenyl groups. In addition, π–π stacking from the layered framework level of the TPE‐Ph‐COF further restricts the rotation of the four phenyl groups. Therefore, the combined locking effects of the intralayer covalent bonds, along with the interlayer noncovalent π–π interactions, achieve the exceptional fluorescence of the TPE‐Ph‐COF.^61^

#### 3.1.3 Applications of Fluorescent POFs in Analytical Chemistry

*Analysis of Nitrobenzene Derivatives*: In consideration of the intensive electron accepting capability of the nitrobenzene derivatives (also known as nitroexplosives), the electron‐rich POFs, based on fluorescence quenching, have been regarded as one of the most promising approaches for fluorescent detection of nitroaromatics.[[qv: 19c]] In 2013, Jiang and co‐workers^20^ synthesized a fluorescent Py‐azine COF through the solvothermal construction of 1,3,6,8‐tetrakis(4‐formylphenyl)pyrene (TFPPy) with hydrazine. Py‐azine COF, with the azine linkage (—C=N—N=C—), was used for the fluorescent detection of some nitroaromatics, such as 2,4,6‐trinitrophenol (TNP), 2,4‐dinitrotoluene (DNT), 2,4‐dinitrophenol (DNP), 2‐nitrotoluene (NT), and 2‐nitrophenol (NP). In particular, the fluorescence of Py‐azine COF solution was quickly quenched by TNP vapor, with a quenching degree of 69% at a relative low concentration (70 ppm) of TNP. Zhang et al.[[qv: 56a]] demonstrated the rapid preparation of a fluorescent triazine‐based COF (SNW‐1, *S*_BET_ = 478 m^2^ g^−1^) through the condensation reaction between melamine and terephthalaldehyde (TPA) using a microwave‐assisted method. SNW‐1 showed a quick response to some nitroaromatics, such as TNP, 2,4,6‐trinitrotoluene (TNT), and 2,4,6‐trinitrophenylmethylnitramine (tetryl), as these electron‐withdrawing acceptors could cause dynamic fluorescence quenching to the luminescent triazine network donor in solution. Similar to Py‐azine COF,^20^ TNP also led to the strongest fluorescence quenching, as the hydrogen bonds were formed between the hydroxyl of TNP molecules and the secondary amine of SNW‐1. Then, Gomes and Bhaumik[[qv: 56b]] reported the synthesis of a triazine‐functionalized luminescent COF‐TRIPTA (*S*_BET_ = 609 m^2^ g^−1^) based on a Schiff base condensation of Tp and 1,3,5‐tris‐(4‐aminophenyl)triazine (TAPT). Under ultraviolet light irradiation, COF‐TRIPTA in polar solvents possessed a strong luminescence and showed a good detection sensitivity for nitroaromatics through fluorescence quenching, even at concentrations lower than 10^−8^ mol L^−1^. In 2015, Murugavel and co‐workers[[qv: 23a]] synthesized four β‐ketoenamine or imine linked fluorescent COFs, including TAPB‐TFP (*S*_BET_ = 567.0 m^2^ g^−1^), *i*PrTAPB‐TFP (*S*_BET_ = 765.0 m^2^ g^−1^), TAPB‐TFPB (*S*_BET_ = 229.4 m^2^ g^−1^), and *i*PrTAPB‐TFPB (*S*_BET_ = 390.6 m^2^ g^−1^). These COFs, particularly TAPB‐TFPB, with the inherent fluorescence of triphenylbenzene unit, showed high CO_2_ adsorption capacity, as well as fluorescent detection ability for nitroaromatics including TNP, DNT, and *p*‐ or *m*‐dinitrobenzene (*p*‐ or *m*‐DNB).

**Figure 6:**
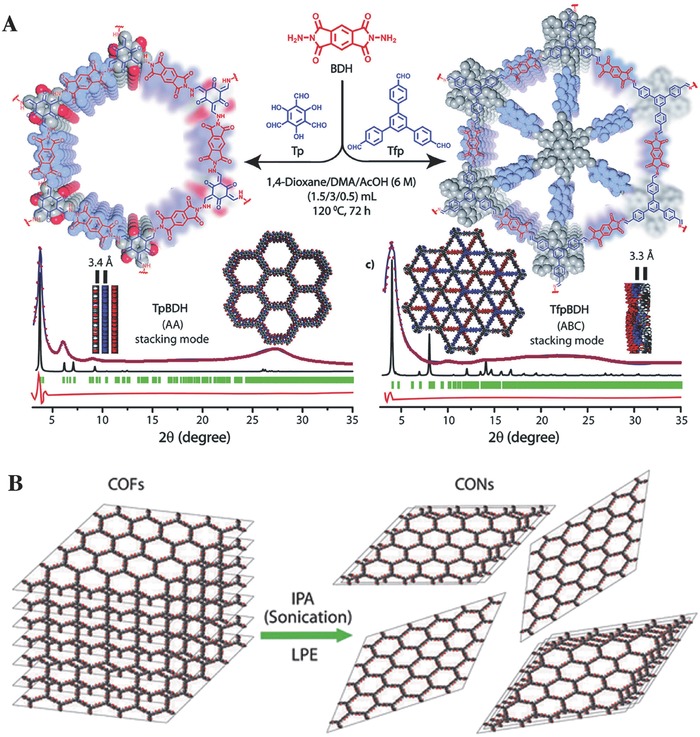
A) A schematic representation for the synthesis of TpBDH and TfpBDH, and the experimental PXRD patterns (blue) compared with simulated (eclipsed; black) and Pawley refined difference between experimental and calculated data (red). B) Schematics of COFs to CONs formation using the liquid phase exfoliation method. Adapted with permission.[[qv: 64c]] Copyright 2015, The Royal Society of Chemistry.

Banerjee and co‐workers[[qv: 64c]] reported the fabrication of thin layered covalent organic nanosheets (CONs, *S*_BET_ = 270 m^2^ g^−1^) derived from the imide linked COF‐TfpBDH through a liquid phase exfoliation approach (**Figure** **6**). TfpBDH‐CONs generated an enhanced luminescence with the irradiation of ultraviolet light than their bulk counterparts (TfpBDH‐COFs). TfpBDH‐CONs displayed an interesting “turn‐on” fluorescent sensing of TNP in their solid state, but exhibited a “turn‐off” mode in their dispersion state. The fluorescence of TfpBDH‐CONs in isopropyl alcohol was quickly quenched by TNP, with an efficient quenching degree of ≈63% at a concentration of TNP as low as 5.4 × 10^−5^ mol L^−1^ (M), and this quenching degree was higher than that of other nitroaromatics, such as TNT, DNT, DNP, and NP. The theoretical calculations further indicated that the electrons of ground‐state transfer from the highest occupied molecular orbital (HOMO) of TNP^−^ to the LUMO of protonated TfpBDH‐CONs, resulting in the observation of the fluorescence quenching. Zhang et al.^65^ also fabricated thin‐layered fluorescent polyimide‐based CONs (PI‐CONs) from a solvothermal‐synthesized COFs through a ultrasonic‐assisted exfoliation method. The PI‐CONs were subsequently used for the fluorescent detection of TNP, demonstrating an excellent chemosensing ability for TNP, based on fluorescence quenching. The density functional theory (DFT) calculations, together with fluorescence lifetime investigations, indicated that fluorescence quenching originating from the combination effects of a) the ground‐state electrons were transferred from the picrate anion (TNP^−^) to PI‐CONs and b) inner filter effect mechanisms (the spectrum overlaps between TNP and PI‐CONs).

Apart from the 2D fluorescent COFs, a 3D pyrene‐based COF (3D‐Py‐COF, *S*_BET_ = 1290 m^2^ g^−1^) with a twofold interpenetrated *pts* topology was synthesized by Lin et al.[[qv: 19c]] This 3D‐Py‐COF, starting from TFFPy and tetra(*p*‐amino‐phenyl)methane (TAPM) monomers, showed the selective adsorption of carbon dioxide over nitrogen and featured a strong yellow‐green fluorescence, which was attributed to the imine‐modified photoelectric pyrene units in their 3D framework. The fluorescence of this 3D‐COF was efficiently quenched by TNP and the quenching degrees reached to 75%, as the TNP amount was 20 ppm.

**Figure 7:**
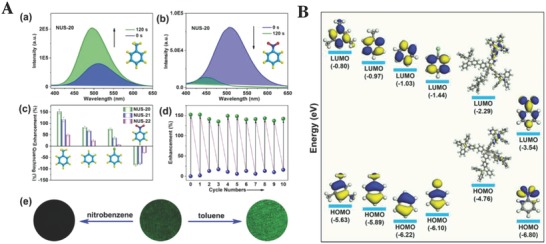
A) Fluorescence emission spectra of NUS‐20 before and after exposure to a) toluene vapor or b) nitrobenzene vapor for 2 min. c) Percentage of fluorescence enhancement or quenching after exposing POFs to different arene vapors for 2 min at 298 K. d) Cycling test of NUS‐20 for the chemical sensing of toluene vapor. e) Fluorescence microscopy images of NUS‐20 before (middle) and after exposure to nitrobenzene (left) or toluene (right) vapors (λ_ex_ = 365 nm). B) HOMO–LUMO energy profiles of mesitylene, toluene, benzene, chlorobenzene, NUS‐20 fragment, and nitrobenzene (from left to right). The difference in the LUMO energy state between NUS‐20 fragment and various VOC analytes [Δ*E*_LUMO_ = *E*_LUMO_ (arene vapors) – *E*_LUMO_ (NUS‐20)] is 1.49, 1.32, 1.26, 0.85, and −1.25 eV for mesitylene, toluene, benzene, chlorobenzene, and nitrobenzene, respectively. Adapted with permission.^67^ Copyright 2016, American Chemical Society.

*Analysis of Small Molecules—Arenes*: In 2015, Xie et al.^66^ synthesized two new 2D COFs, TAT‐COF‐1, and TAT‐COF‐2 (*S*_BET_ = 1166 m^2^ g^−1^), based on the solvothermal condensation of triazatruxene (TAT) derivatives. These two fluorescent COFs, TAT‐COF‐2 in particular, have moderate adsorption capacities of hydrogen (109.26 cm^3^ g^−1^) and carbon dioxide (38.99 cm^3^ g^−1^), good selectivity of CO_2_/N_2_ (≈5.9), and fluorescence emission ability. The fluorescence intensity of TAT‐COF‐2 can be strengthened by some arenes that are rich in electrons (e.g., toluene, chlorobenzene, *o*‐dichlorobenzene, mesitylene, bromobenzene, and 1,2‐dimethylbenzene) but are, conversely, quenched by the electron deficient arenes (nitrobenzene and 2‐nitrotoluene). This phenomenon was attributed to the opposite directions of electron transfer: the fluorescence of TAT‐COF‐2 was enhanced in the presence of some arenes with abundant electrons because the electron cloud density of this COF will be strengthened, due to the electrons transferred from these electron‐donating arenes. By contrast, some electron‐withdrawing arenes reduce the electron cloud density of TAT‐COF‐2, resulting in the appearance of fluorescence quenching. Furthermore, after removing these electron‐donating arenes, the previously enhanced fluorescence intensity takes only a few minutes to recover back to the normal level because the volatile arenes can easily escape from the frameworks of TAT‐COF‐2, which indicated that TAT‐COF‐2 can be conveniently reused for the detection of electron rich arenes. Dong et al.^67^ reported constructing a series of POFs (NUS‐20–NUS‐23, *S*_BET_ = 368–900 m^2^ g^−1^) based on flexible AIE‐active TPE units as molecular rotors through Suzuki–Miyaura or Sonogashira–Hagihara reactions. The POFs presented noticeably “turn‐on” or “turn‐off” fluorescence emission upon exposure to various arenes (also known as volatile organic compounds, VOCs). Similar to TAT‐COF‐2, the size‐selective “turn‐on” fluorescence enhancement was induced by the electron‐rich arenes (e.g., toluene, **Figure** **7**A) while the “turn‐off” fluorescence quenching was achieved for chemical sensing of the electron‐deficient arenes (e.g., nitrobenzene, Figure 7A). Furthermore, the DFT calculations (Figure 7B) indicated that the electrons can transfer from the LUMO of NUS‐20 (−2.29 eV) to that of nitrobenzene (−3.54 eV), contributing to the fluorescence quenching. However, the electrons conversely transferred from the LUMO of electron‐rich arenes, such as mesitylene (0.80 eV), toluene (0.97 eV), benzene (1.03 eV), and chlorobenzene (1.44 eV), to NUS‐20, therefore, fluorescence enhancement was observed.

*Analysis of Small Molecules—Ammonia*: As mentioned earlier in Section 3.1.2,^61^ the TPE‐cored boronic acids were incorporated with THB monomer to synthesis a luminescent TPE‐Ph‐COF with the columnar AIE π‐arrays. As the fluorescence microscope images shown in Figure 5B, all as‐synthesized TPE‐Ph‐COF from different reaction times (3–30 days) demonstrated brilliant luminescence of the same color, indicating that the structural growth of TPE‐Ph‐COF does not alter its luminescent characteristics. TPE‐Ph‐COF was further explored as a highly sensitive fluorescence sensor for NH_3_, with a fluorescence quenching rate constant (*k*_q_) of 6.3 × 10^14^ m ^−1^ s^−1^ in toluene and 1.4 × 10^14^ m ^−1^ s^−1^ in cyclohexane (Figure 5C). The high *k*_q_ values revealed that TPE‐Ph‐COF has an excellent fluorescence detection ability for ammonia.

*Analysis of Small Molecules—Water*: Yan and co‐workers^68^ designed an imine‐linked COF, TzDa (*S*_BET_ = 709 m^2^ g^−1^) through the condensation reaction between 4,4′,4″‐(1,3,5‐Triazine‐2,4,6‐triyl)trianiline (Tz) and 2,5‐dihydroxyterephthalaldehyde (Da). The prepared mesoporous COF‐TzDa (pore size, ≈3.6 nm) in organic solvents interestingly exhibits doubled fluorescence spectrums: the first fluorescent wavelength of ≈500 nm was primarily attributed to electrons transfer in the intramolecular of Tz units from phenyl groups (donor) to triazine rings (acceptor); another wavelength at 590 nm was originated from the excited‐state intramolecular proton transfer effect, based on the interactions of hydrogen bonds between O—H of Da units and newly formed imine‐bonds of TzDa. Moreover, the highly sensitive fluorescent emissions guarantee TzDa as a potential fluorescent sensor for the detection of water from organic solvents, with a rapid response (within 1 min), low limit of detection (0.006%), acceptable repeatability (relative standard deviations (RSDs) 0.03–0.24%, *n* = 10), as well as good reusability (8 recycling detections, RSDs < 4.1%).

*Analysis of Small Molecules—pH Sensor in Water*: Zhang et al.^69^ reported for the construction of a β‐ketoenamine based COF‐JLU4 (*S*_BET_ = 923 m^2^ g^−1^) through the solvothermal condensation between Tp and 2,5‐dimethoxyterephthalohydrazide. COF‐JLU4 acted as a fluorescent pH sensor in water solution, with the advantages of being highly crystalline and having intensive fluorescence, as well as aqueous wettability. The fluorescence spectrum of this COF was closely related to the pH values of water solution: the strongest fluorescence intensity originated from the most acidic solution (pH 0.9) and the weakest fluorescence from the basic solution at pH 13.0. Moreover, the fluorescence intensity of COF‐JLU4 was gradually reduced, as the pH values increased from 9.0 to 13.0, which resulted from the transversion of deprotonation in the N—H bonds from the frameworks.

*Analysis of Metal Ions—Hg^2+^*: Mercury is a deleterious heavy metal that can cause functional disorders of organs and the central nervous system.^70^ Accordingly, considerable efforts are being made to develop new methods based on fluorescent adsorbents for the detection of mercury ions. Some porous materials, such as hydrogels, silicas, and mesoporous carbons, usually have limited capacities.^71^ Although MOFs^72^ have been used for mercury (II) removal based on their high surface areas, their stable performance in aqueous solutions remains challenging. Meanwhile, POFs with predesigned porous architectures render them attractive as a molecular platform for the effective detection and removal of Hg^2+^.^73^ Ding and co‐workers[[qv: 73a]] synthesized a thioether‐modified fluorescent COF‐LZU8 (*S*_BET_ = 473 m^2^ g^−1^) for the sensitive and selective detection of the toxic mercury (II). The fluorescence detection of Hg^2+^ by COF‐LZU8 was based on the “turn off” mode: Hg^2+^ was first unsaturated and coordinated to the thioether groups of COFs, and then electrons transferred from COF‐LZU8 to the unoccupied d‐orbitals of Hg^2+^, which resulted in significant fluorescence quenching. The inductively coupled plasma (ICP) analysis revealed that higher than 80% of fluorescence could be quenched by only a low content (11.6%) of Hg^2+^, attributed to the special architecture of COF‐LZU8: one unit cell of COF‐LZU8 contains six thioether groups, which can form six of Hg—S coordinate bonds to efficiently quench fluorescence. Compared with other competitive metal ions (e.g., Li^+^, Na^+^, Co^2+^, K^+^, Mg^2+^, Cu^2+^, Al^3+^, Fe^2+^, Fe^3+^, etc.), only Hg(II) led to a dramatic fluorescence quenching of COF‐LZU8, and none of the metal ions had a perceptible interferential effect on mercury, indicating that COF‐LZU8 has an intensive detection selectivity toward Hg^2+^. Jiang and co‐workers[[qv: 73b]] constructed a methyl sulfide groups‐functionalized imine‐COF, TAPB‐BMTTPA‐COF (*S*_BET_ = 1934 m^2^ g^−1^), by employing TAPB as a knot and 2,5‐bis(methylthio)terephthalaldehyde (BMTTPA) as a linker. TAPB‐BMTTPA‐COF exhibited a highly effective adsorption capacity for Hg(II) due to the interactions between Hg^2+^ and the methyl sulfide groups from TAPB‐BMTTPA‐COF, which led to the electrons transfer from the COF to Hg^2+^ and then triggered fluorescence quenching. At approximately the same time, a vinyl‐functionalized COF (COF‐V) precursor was first designed by Sun and co‐workers^74^ through the condensation of 1,3,5‐tris(4‐aminophenyl)‐benzene (Tab) and 2,5‐divinylterephthalaldehyde (Dva). The sulfur‐based COF (COF‐S‐SH, *S*_BET_ = 546 m^2^ g^−1^) was then fabricated through the chemical modification of COF‐V with a sulfur derivative (1,2‐ethanedithiol), based on a thiol‐ene “click” reaction. This COF showed a high Hg^2+^ and Hg^0^ adsorption capacity, with the selective reduction of Hg^2+^ from 5 ppm to a relatively low level of 0.1 ppb in aqueous solution, much lower than the United States Environmental Protection Agency (USEPA) limits of mercury in drinking water (2 ppb).

*Analysis of Metal Ions—UO_2_^2+^*: Uranium is an essential resource of nuclear energy with radioactivity and biological toxicity, therefore, preconcentration and detection of uranium from the nuclear industrial waters has stimulated increasing concern.^75^ Ma and co‐workers[[qv: 34b]] constructed a 2D thin nanosheet of fluorescent COF (NS‐COF) at the interface of two miscible organic solvents via an interesting buffering interlayer interface approach. The newly synthesized NS‐COF, which has the benefits of good thermal and solvent stability, exhibits a selective and sensitive adsorption capacity toward UO_2_^2+^ from simulated industrial solutions. As the addition amount of UO_2_^2+^ increased, the fluorescence intensities of this COF gradually decreased in dimethylformamide (DMF) solution. NS‐COF still showed a strong fluorescence response, even if the concentration of uranyl (IV) ions was lower than 0.2 ppm. The fluorescence of NS‐COF was nearly entirely quenched, as the concentration of UO_2_^2+^ increased to 8.6 ppm.

*Analysis of Metal Ions—Cu^2+^*: Li et al.^76^ synthesized a H‐bonding azine‐based fluorescent COF‐JLU3 (*S*_BET_ = 570 m^2^ g^−1^) through the condensation comonomers of hydrazine hydrate and 1,3,5‐tris(3′‐*tert*‐butyl‐4′‐hydroxy‐5′‐formylphenyl)benzene, using an acetic acid catalyst. Interestingly, the fluorescence‐emission of COF‐JLU3 was interrelated with the types of metal ions: the transition metal ions with filled d‐orbitals (e.g., Zn^2+^, Cd^2+^, Pb^2+^, and Ag^+^), alkaline metal (Li^+^, Na^+^, and K^+^), and alkaline‐earth (Mg^2+^, Ca^2+^, and Ba^2+^) scarcely demonstrated any influence on their fluorescence intensity, however, the metal ions with unoccupied d‐orbitals such as Fe^3+^, Co^2+^, Ni^2+^, and Cu^2+^ can quench the fluorescence of COF‐JLU3 at different levels. In particular, Cu^2+^ showed the strongest effect on fluorescence quenching, as considerably active sites on the pore wall of COF‐JLU3, such as N atoms and —OH groups, can effectively be coordinated with copper ion, leading to the electron transfer from COF‐JLU3 to copper (II).

*Analysis of Metal Ions—Fe^3+^*: Recently, Wang et al.^77^ constructed two polyimide‐based COFs, PI‐COF‐201 and PI‐COF‐202, using a fast direct heating method. The large π‐conjugation delocalization and inherent rigid architectures are two primary causes for the generation of intensive fluorescence. These PI‐COF can be utilized as a fluorescence sensor for Fe^3+^, based on the “turn‐off” mode, and the fluorescence quenching quality of PI‐COFs was attributed to the electrons transfer from these COFs to the unoccupied d‐orbital of Fe^3+^. Liao and co‐workers^78^ prepared microporous polyimide (PI) networks utilizing melamine and perylene derivatives as comonomers, with a Lewis acid catalyst. The PI networks showed an intensive yellow‐green fluorescence in tetrahydrofuran (THF) dispersions, and some metal ions, such as Cu^2+^, Fe^2+^, Co^2+^, Mn^2+^, Pb^2+^, and Al^3+^, can decrease 15–20% of their fluorescence intensities. In particular, Fe^3+^ possesses a higher electron affinity compared with the above metal ions, resulting in a stronger fluorescence quenching (56%).

### 3.2 Chromatographic Separation

In analytical chemistry, chromatography plays an important role in the separation and determination of multiple analytes from complex samples. By virtue of the interaction between analytes and the stationary phase in a chromatographic column, the mixture of analytes can be efficiently separated into simple individual components. In recent years, considerable advanced materials have been utilized as the innovative stationary phases for chromatographic separation. Because of their distinguishing characteristics, POFs have been regarded as potential candidates for novel stationary phases for chromatographic separation systems, including GC, HPLC, and CEC. The chromatographic separation mechanism depends on the molecular sieving effect,^79^ van der Waals,[[qv: 33b]] hydrogen bonds,[[qv: 33b,80]] π–π,[[qv: 33b,80]] and hydrophobic[[qv: 80a]] interactions between the POFs‐based stationary phases and the analytes of interest. In this part, we specifically focus on the recent advancements of POFs as the stationary phases for GC, HPLC, and CEC.

#### 3.2.1 Gas Chromatography

GC, a crucial and extensive used separation technique, serves as an essential part in the analysis of volatile organic compounds. The GC stationary phases, coated on a fused silica capillary as a chromatographic column, are continuously being developed to enhance its separation capabilities. Considering the limitations of conventional stationary phases, such as nonpolar dimethylpolysiloxane or polar polyethylene glycol, many researchers have tried to create novel stationary phases with more thermal stability and/or greater selectivity. As a class of microporous crystalline hybrid materials, MOFs^81^ have been investigated for GC separations of various analytes. However, the chromatographic performance of some MOFs was not satisfactory, due to their wide particle size distributions and low stability.[[qv: 81e]] Compared with MOFs, POFs with unique characteristics, such as high surface area, lower density, and high stability, have become the potential stationary phases for chromatographic separations with high efficiency.

**Figure 8:**
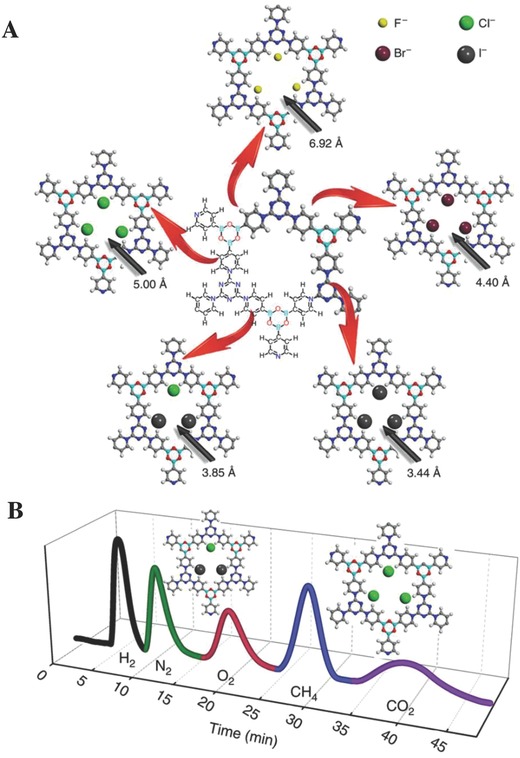
A) A schematic diagram for the preparation of F‐PAF‐50, Br‐PAF‐50, 2I‐PAF‐50, and 3I‐PAF‐50 from Cl‐PAF‐50. B) GC chromatograms for separation of H_2_, N_2_, O_2_, CH_4_, and CO_2_ mixture using the connective column combined Cl‐PAF‐50 with 2I‐PAF‐50 stationary phases. Adapted with permission.^79^ Copyright 2014, Macmillan Publishers Limited.

In 2014, Zhu and co‐workers^79^ pioneered to synthesize a series of quaternary pyridinium‐type PAFs (X‐PAF‐50, X = F, Cl, Br, and I, *S*_BET_ = 96–614 m^2^ g^−1^) with tailor‐made pore sizes (3.4–7 Å) using a feasible ion exchange method (**Figure** **8**A). The tunable pore sizes of X‐PAF‐50 offer the benefits to capture or sieve gas molecules with different sizes based on the molecular sieving effects. Five gas mixture (H_2_, N_2_, O_2_, CH_4_, and CO_2_) can be efficiently separated by gas chromatography on the connective columns of Cl‐PAF‐50 and 2I‐PAF‐50 as stationary phases (Figure 8B). Later, Yan and co‐workers[[qv: 33b]] developed an easy room temperature method for the rapid synthesis of spherical COF‐TpBd (*S*_BET_ = 885 m^2^ g^−1^) within 30 min. Because of the large surface area, high solvent stability (in organic solvents, water, HCl, or NaOH), and good thermostability (up to 250 °C), COF‐TpBd was selected for the GC stationary phase. This COF‐TpBd coated capillary column showed satisfactory baseline GC separations of various targets, such as *n*‐alkanes, pinene isomers, alcohols, benzene, and cyclohexane. Their results demonstrated the great potential of POFs in the chromatographic stationary phases for GC.

Although considerable POFs with diverse features have been developed, the design and synthesis of chiral POFs with stable crystallinity are still rarely exploited because of the extremely limited availability of chiral POFs and their fabrication challenges,^82^ which prevents their further practical utilization in chromatographic separation.^83^ Dong and co‐workers[[qv: 82a]] synthesized a series of chiral diene‐based POFs (POF‐1, POF‐2, and POF‐3, *S*_BET_ = 252–471 m^2^ g^−1^) via a cross‐coupling polycondensation. The as‐synthesized POF‐1 was demonstrated for use as a novel catalyst for the asymmetric conjugation addition reactions, and moreover, as a stationary phase for chiral gas chromatographic separation of 1‐phenylpropanol and 1‐phenylpropylamine racemics. Yan and co‐workers^83^ developed a bottom‐up approach to synthesize three β‑ketoenamine linked chiral COFs, CTpPa‐1, CTpPa‐2, and CTpBd (*S*_BET_ = 104–317 m^2^ g^−1^) and then in situ fabricated these chiral COFs onto the capillary columns for GC. For the synthesis of these chiral COFs, the Tp monomer was first functionalized with chiral (+)‐diacetyl‐l‐tartaric anhydride to obtain a chiral modified monomer‐CTp, then another monomer, such as Pa‐1, Pa‐2, and BD, is ploycondensed with CTp to synthesize chiral CTpPa‐1, CTpPa‐2, and CTpBd, respectively. For the fabrication of chiral COFs coated capillary columns, the prepolymerization chiral COFs dispersion was filled into a 3‐aminopropyltriethoxysilane (APTES) modified fused‐silica capillary and completed condensation reaction at 80 °C for 4 h. The prepared chiral COFs coated capillary columns exhibited the baseline separation of enantiomers, with good repeatability (RSDs < 1.89%) and reproducibility (RSDs < 3.41%). These studies promoted the preparation of chiral POFs and broaden their use in chiral chromatographic separation.

#### 3.2.2 High‐Performance Liquid Chromatography

The universal relevance and high analytical precision makes HPLC an advanced separation technique in the areas of chemical, pharmaceutical, food, environment sciences, and so on. The core component of HPLC is the chromatographic column packed with an appropriate stationary phase to achieve a high resolution efficiency. There has been an unceasingly accelerating progression of the stationary phases for HPLC under the further interdisciplinary development of innovative materials. Given the remarkable features of POFs, they should be promising stationary phase materials for HPLC. However, the direct packing of the POFs into HPLC columns might cause the troubles of high column back‐pressure, as well as low column efficiency,^84^ as the POFs prepared by the traditional approaches often suffered from some defects, such as sub‐micrometer size, irregular shape, or broad size distribution.^84^ To address these issues, one method is to incorporate POFs into monolithic columns, while another one is to assemble POFs onto SiO_2_ microspheres for the fabrication of core–shell microspheres packed HPLC columns.^85^

As the first choice, Yan and co‐workers[[qv: 84b]] synthesized a COFs‐based monolithic column to promote HPLC performance on the separation of some organic analytes. This COFs‐based column, termed a poly (TpPa‐MA‐co‐EDMA) monolithic column (*S*_BET_ = 224 m^2^ g^−1^), was prepared through the condensation of methacrylate‐modified COF TpPa‐1 with a polymerization precursor. This monolithic column demonstrated good baseline separation of polycyclic aromatic hydrocarbons (PAHs), anilines, phenols, benzothiophenes, and nonsteroidal anti‐inflammatory drugs (NSAIDs), with high column efficiency and acceptable precisions. As another method to overcome the restrictions on the application of POFs in HPLC stationary phases, Zhao et al.[[qv: 80b]] have fabricated a hybrid CTF‐1@SiO_2_ (*S*_BET_ = 359 m^2^ g^−1^) stationary phase packed columns for the efficient HPLC separation of diverse analytes, including PAHs, monosubstituted benzenes, phenols, anilines, and bases. The CTF‐1@SiO_2_ microspheres were prepared through the regular trimerization of the terephthalonitrile monomer to grow CTF‐1 onto the surface of cyano‐functionalized silica sphere. Because of the synergistic effects from the improved separation ability of CTF‐1 frameworks and the good column packing property of the silica spheres, better separation selectivity was achieved on this CTF‐1@SiO_2_ packed HPLC column than the CN‐SiO_2_ or C_18_ columns. Yan and co‐workers[[qv: 80a]] developed an in situ growth strategy to fabricate a core–shell structured COF‐TpBd@SiO_2_ composite (*S*_BET_ = 385 m^2^ g^−1^) with a uniform and controllable TpBD shell. Subsequently, the COF‐TpBd@SiO_2_ microspheres were packed into a chromatographic column as the HPLC stationary phase for the efficient separation of acidic (hydroquinone, *p*‐cresol, and *p*‐chlorophenol), neutral (PAHs, toluene, and ethylbenzene), and basic (*n*‐phenylacetamide, 4‐methylaniline, and *p*‐nitroaniline) analytes. The COFs@SiO_2_ composites‐packed HPLC columns showed high resolution and good column efficiency due to the interactions of π–π, hydrophobic, and the hydrogen bonds between the COFs@SiO_2_ composites and the analytes. In addition, some basic components in living cells, such as nucleobases, nucleosides, deoxynucleosides, 5 mdC, and dC, can also be easily and directly baseline‐separated under the isocratic elution.

**Figure 9:**
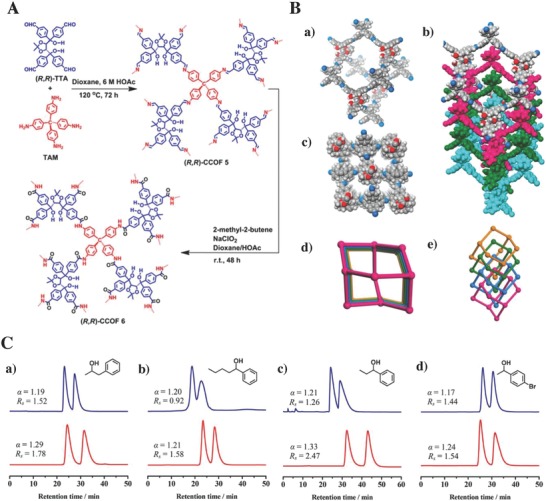
A) The synthesis of chiral CCOF‐5 and CCOF‐6. B) Structural representations of CCOF‐5: a) single diamondoid network, b,c) four‐interpenetrated diamond nets along *a*‐axis and *c*‐axis, and d,e) structural models of fourfold interpenetrated diamond nets. C‐gray, N‐blue, H‐white, and O‐red. C) The chromatographic separation of racemic molecules: a) 1‐phenyl‐2‐propanol, b) 1‐phenyl‐1‐pentanol, c) 1‐phenyl‐1‐propanol, and d) 1‐(4‐bromophenyl)ethanol by using the CCOF‐5 (upper blue line) and CCOF‐6 (lower red line) packed HPLC columns, respectively. Adapted with permission.[[qv: 25b]] Copyright 2018, American Chemical Society.

Similar to GC, some chiral POFs can also be used as the novel HPLC stationary phases for chiral separation. Recently, Zhang and co‐workers^86^ synthesized a hydrazone‐based chiral COF, BtaMth‐COF (*S*_BET_ = 723 m^2^ g^−1^), based on a bottom‐up strategy, and then the BtaMth‐COF was constructed onto the supporting silica spheres to form BtaMth‐COF@SiO_2_ composites using a one‐pot synthesis method. Its high crystallinity and good chemical stability make BtaMth‐COF@SiO_2_ a good match for the chiral HPLC stationary phase for high‐resolution separation of positional isomers, such as nitrochlorobenzene and nitrotoluene, in the reverse‐phase mode, while *cis*–*trans* isomers, including metconazole and β‐cypermethrin, are good for the normal‐phase mode. Han and co‐workers[[qv: 25b]] designed a bottom‐up approach to prepare a 3D chiral imine‐linked CCOF‐5 (*S*_BET_ = 655 m^2^ g^−1^) through the construction of tetrahedral tetra(4‐anilyl)methane and chiral tetraaryl‐1,3‐dioxolane‐4,5‐dimethanols‐modified tetraaldehyde (R,R′‐TTA). Another 3D chiral amide‐linked COF, CCOF‐6 (*S*_BET_ = 613 m^2^ g^−1^), was then constructed through the postsynthetic oxidation of the imine linkages from the frameworks of CCOF‐5, with a similar crystallinity and porosity to CCOF‐5 but an improved chemical stability (**Figure** **9**A). Both the chiral COFs showed a fourfold interpenetrated diamondoid open‐pore architecture embellished with chiral dihydroxy groups (Figure 9B), which laid the structural foundation to act as a chiral stationary phase for HPLC. Compared with the imine‐linked CCOF‐5, the oxidized CCOF‐6 packed column achieved better HPLC separation ability for racemic alcohols (Figure 9C) and other chiral molecules. These studies revealed the potential candidates of the chiral POFs as a novel stationary phase for the chiral separations.

#### 3.2.3 Capillary Electrochromatography

As a hybrid electroseparation technique, CEC inherits the synergy merits of the selectivity from HPLC and the separation efficiency from capillary electrophoresis (CE). In CEC, the electroseparation is applied based on the electrophoretic mobility of the analytes and their partitioning between the stationary phase coated on the inner wall of a fused silica capillary and mobile buffer phase. Open‐tubular CEC (OT‐CEC), which is recognized as an alternative mode of CEC, has recently attracted increasing attention due to its prominent advantages, such as good permeability and facile capillary fabrication, particularly accessible to develop new materials as stationary phase. However, OT‐CEC still suffers from some problems, such as a relatively low sample capacity and an inadequate phase ratio,[[qv: 84a,87]] which slows down the further development of this approach. Considering the unique properties of POFs, it is possible to choose POFs as a novel stationary phase material for OT‐CEC columns to diminish the previously mentioned shortcomings.^88^

Niu et al.[[qv: 88a]] pioneered to synthesize an imine‐based COF‐LZU1 as a novel stationary phase for OT‐CEC. The COF‐LZU1 coated OT‐capillary was fabricated through a covalent bonding method by filling the dispersion of COF‐LZU1 into a 3‐glycidoxypropyltrimethoxysilane (GLYMO)‐functionalized capillary, followed by thermal treatment at 70 °C for 4 h. The formation of chemical bonds between the amino groups of COF‐LZU1 and epoxy groups of GLYMO ensured the high stability of COFs‐assisted stationary phase. This COF‐LZU1‐coated capillary obtained satisfactory baseline separations of alkylbenzenes, PAHs, and anilines, due to the molecular sieving effect of COF‐LZU1 as well as the hydrophobic interactions between COFs and analytes. Furthermore, the developed OT‐CEC method, based on the COF‐LZU1‐coated capillary, had a large load capacity (naphthalene, 0.6 mg mL^−1^), good stability (>300 runs), and acceptable precision (intraday RSDs < 8.7%). At nearly the same moment, Chen and co‐workers[[qv: 88b]] reported taking a polydopamine‐supported immobilization approach, along with a layer‐by‐layer growth method, to prepare a multilayer COF‐5 coated capillary. The as‐fabricated capillary was then applied to OT‐CEC separate of neutral, acidic, and basic molecules with the successful baseline separations. This multilayer COFs‐assisted capillary demonstrated a high resolution and column efficiency (e.g., 1.5 × 10^5^ theoretical plates m^−1^ for methylbenzene) and good stability, as well as acceptable repeatability (RSDs < 5%). Recently, using APTES and glutaraldehyde as a cross‐linker, COF‐LZU1 was also in situ synthesized and epitaxial grafted on a capillary column for OT‐CEC by Chen and co‐workers.[[qv: 88c]] The π–π, hydrophobic, and a certain degree of hydrophilic interaction of COF‐LZU1 resulted in the baseline separations of neutral analytes, amino acids, and NSAIDs. Compared with the previous method of Niu et al.,[[qv: 88a]] this in situ synthesis method is facile, which avoided the independent synthesis process. The COF‐LZU1 coated OT‐capillary possessed the acceptable intraday (RSDs < 1.6%), interday (<5.6%), column‐to‐column (<6.8%) repeatability and good stability after being flushed with ethanol for 20 h or consecutive CEC separations of more than 60 times. Ye et al.[[qv: 88d]] fabricated a COF‐SNW‐1‐coated capillary column through a covalent bonding reaction between SNW‐1 and APTES‐modified capillary inner walls. The prepared COF‐SNW‐1 column was used for the OT‐CEC separation of sulfonamides, cephalosporins, amino acids, and parabens, with good separation efficiency and reproducibility. Additionally, to the best of our knowledge, although there are (to date) no reports on the utilization of COFs, CTFs, or PAFs as the chiral stationary phases for CEC, Zhang and co‐workers^89^ prepared a chiral porous organic cage (CC3‐R)‐coated capillary in OT‐CEC for the selective separation of chiral analytes and positional isomers, which was an inspiration for the synthesis of suitable POFs in chiral CEC separations.

### 3.3 Sample Preparation Methods

Sample preparation, also referred to as sample pretreatment (primarily used for the enrichment of target analytes but the simultaneous exclusion of interfering matrix components), is an essential process in separation science and plays a critical role in analytical chemistry. In most cases, sample preparation still represents the “bottleneck” in the pursuit of optimum analytical methods, particularly for the detection of trace‐level compounds from the complex matrices. Currently, there is a trend toward the exploration of novel porous materials in the sample preparation process, including sampling, clean‐up, preconcentration, and separation, as the characteristics of these porous materials are significant to the sensitivity, selectivity, and repeatability of sample preparation techniques. The purpose of this section is to show solicitude for the potential application of COFs, CTFs, and PAFs as advanced materials for the effective extraction of diverse analytes from various samples.

#### 3.3.1 Solid Phase Extraction

SPE, as a beneficial alternative sample pretreatment method to liquid–liquid extraction (LLE), has been preferably used for the preconcentration of analytes from diverse sample matrices.^90^ The utilization of SPE can efficiently avoid some shortcomings of LLE, such as large volumes of organic solvent consumption, difficult to complete phase separation, and time‐consuming features.^90^ Recently, much effort has been directed toward the consummate SPE technique, with the help of novel adsorbents with improved selectivity and enhanced adsorptive capacity, to meet the specific requirements of analytical chemistry.[[qv: 90b]]

In 2016, Hu and co‐workers^91^ adopted CTF‐1 as an adsorbent for on‐line SPE of three nitroimidazoles from water and porcine liver samples through flow injection, followed by HPLC‐UV detection. The CTF‐1 SPE sorbents exhibited high extraction efficiency toward nitroimidazoles, due to π–π conjugation and intermolecular hydrogen bonds. Liu et al.^92^ reported another example of an on‐line SPE system, based on COF‐CTpBd (*S*_BET_ = 114 m^2^ g^−1^) adsorbents for SPE of some heavy metal ions from milk and aqueous samples. COF‐CTpBd was filled into a PTFE microcolumn to fabricate an on‐line SPE cartridge. Combined with the inductively coupled plasma mass spectrometry (ICP‐MS) detection, this COF‐assisted on‐line SPE method has achieved wide linear ranges (0.05–25 µg L^−1^) and low limits of detection (LODs, 2.1–21.6 ng L^−1^) for the target ions. Zhang and co‐workers^93^ reported the synthesis of a hydrazine‐based covalent organic polymer (HL‐COP) by the condensation of TPA and 1,3,5‐benzenetricarbohydrazide via Schiff‐base chemistry. HL‐COP was used as an on‐line SPE adsorbent, combined with HPLC‐UV detection for preconcentration of Sudan dyes from chili powder and sausage samples, with LODs of 0.03–0.15 µg L^−1^ and enrichment factors of 305–757. Notably, our group^94^ synthesized COF‐TpAzo (*S*_BET_ = 636 m^2^ g^−1^) through an eco‐friendly mechanochemical method, based on the Schiff base aldehyde‐amine condensation of Tp and 4,4′‐azodianiline (Azo). TpAzo was used as the SPE adsorbent for the extraction of benzoylurea insecticides from juice, tomato, and white radish samples with good extraction capabilities. Recently, imine‐linked molecularly imprinted COFs (MICOFs, *S*_BET_ = 432 m^2^ g^−1^) were synthesized by Ji et al.,^95^ who used fenvalerate as a dummy template for SPE of four structurally similar cyano‐pyrethroids, such as fenvalerate, flucythrinate, β‐cyfluthrin, and λ‐cyhalothrin. Coupled with HPLC‐DAD, the developed MICOFs‐SPE method was sensitive and selective, with high recoveries (94.3–102.7%) of cyano‐pyrethroids in vegetable, fruit, and traditional Chinese medicine samples.

#### 3.3.2 Dispersive Microsolid Phase Extraction

The above on‐line SPE systems efficiently reduced the tedious procedural steps, saved analysis time, minimized the exposure to hazardous solvents, and improved sample throughput and reproducibility.^91^ However, only a limited number of POFs^91^, ^92^, ^93^, ^94^ could be directly employed for on‐line SPE systems, as their irregular shapes and nanometer particle diameters might cause high SPE‐column backpressure,[[qv: 84a]] which restricts the further utilization of POFs for on‐line SPE system. D‐µ‐SPE is an alternative SPE approach to avoid the above problems of on‐line SPE. In D‐µ‐SPE, the suspension of nanomaterial adsorbents are directly mixed into the aqueous sample and dispersed by ultrasonication, and then the adsorbents are separated from the sample solution by centrifugation or filtration.^96^

Liu et al.^97^ reported the use of CTF‐1 as adsorbent for D‐µ‐SPE of various aromatic analytes with high adsorption capacity and rapid adsorption/desorption kinetics. Their results revealed that CTF‐1 achieved greater adsorption efficiency toward monocyclic/bicyclic aromatics with substituted groups (e.g., hydroxyl, amino, nitro, and sulfonate) than the nonsubstituted aromatics (e.g., benzene and naphthalene). Some non‐hydrophobic mechanisms were considered to be explanations for the good extraction performance of CTF‐1, such as hydrogen bonding (—OH, —NH_2_‐substituted aromatics), electrostatic attraction (anionized compounds), and π–π interaction (nitroaromatics). CTF‐1 showed a higher adsorption capacity toward the polar and ionic analytes than commercial XAD‐4 adsorbent. In 2015, Ma and co‐workers^98^ constructed a COF‐COOH by condensation of trimesoyl chloride and *p*‐phenylenediamine through a simple one‐step method at room temperature. Then, a new SPE adsorbent, COF‐HBI, was synthesized through the modification of COF‐COOH with 2‐(2,4‐dihydroxyphenyl)‐benzimidazole (HBI). COF‐HBI exhibited excellent selective D‐µ‐SPE ability toward UO_2_^2+^ compared with eleven competing ions from the simulated nuclear industrial effluent. This group also synthesized other COFs,^99^ including CTF‐CCU, CCTU, and CCTS,[[qv: 99a]] carbonaceous COFs,[[qv: 99b]] MP‐COF,[[qv: 99c]] and NS‐COF,[[qv: 34b]] for D‐µ‐SPE of UO_2_^2+^. Li et al.^100^ reported the successful functionalization of PAF‐1 with the uranyl chelating amidoxime group (PAF‐1‐CH_2_AO) for uranium extraction from the simulated seawater samples. The PAF‐1‐CH_2_AO exhibits a high uranium adsorption capacity and can reduce the UO_2_^2+^ concentration from 4.1 ppm to less than 1.0 ppb in water within 90 min. Recently, Zhu and co‐workers^101^ developed a series of molecularly imprinted PAFs (MIPAF‐11s, *S*_BET_ = 95–524 m^2^ g^−1^) using a Heck‐coupling reaction for the selective extraction of uranium (VI) ions from the simulated seawater. MIPAFs exhibited favorable compatibility with some conventional polymers, allowing for facile operational ease and the flexibility to obtain MIPAF‐composite films, fibers, and coatings for practical applications for the selective extraction of UO_2_^2+^ from the interfering ions.

In 2017, Ma and co‐workers^102^ synthesized COF TpPa‐1 as an adsorbent for extraction of *N*‐linked glycopeptides from human serums, coupled to the matrix‐assisted laser desorption ionization‐time of flight‐mass spectrometry (MALDI‐TOF‐MS) detection. The TpPa‐1‐assisted D‐µ‐SPE method displayed high sensitivity at the *fmol*‐level by the selective extraction of glycopeptides from tryptic digests. The affluent binding sites in TpPa‐1 provided this COF with higher extraction capacity (178 mg g^−1^, IgG/TpPa‐1) and good reusability (>10 times). Wang et al.^103^ constructed a flower‐shaped COF‐TpPa‐2‐Ti^4+^, with a regular nano‐hierarchical structure by chelating titanium (IV) ions into COF‐TpPa‐2 frameworks. The as‐synthesized COF‐TpPa‐2‐Ti^4+^ exhibited a low limit of detection (4 *fmol*), high extraction capacity (100 µg mg^−1^), and good selectivity (β‐casein: BSA = 1: 100) for phosphopeptide preconcentration from β‐casein. Additionally, COF‐TpPa‐2‐Ti^4+^ showed large extraction ability toward phosphopeptide from other complex sample matrices.

#### 3.3.3 Magnetic Solid‐Phase Extraction

As another innovative pattern of SPE, MSPE, based on the use of magnetic adsorbents, was demonstrated as a promising miniaturized method for the enrichment and separation of diverse analytes from large sample volumes.^104^ In MSPE, the magnetic adsorbents are directly dispersed into sample solutions promoting analyte adsorption by vortex, oscillation, or sonication. Then, the magnetic adsorbents are facilely disengaged from the sample solutions, with the assistance of an external magnet and without the need for centrifugation or filtration like D‐µ‐SPE, which efficiently simplifies the extraction process.[[qv: 104e]] In addition, the magnetic adsorbents can be easily recycled and reused, which is cost effective and environmentally friendly. To date, a considerable number of MSPE adsorbents have been prepared using the following two methods: I) the direct use of magnetic nanoparticles (MNPs), which are primarily composed of iron, nickel, cobalt, or any of their oxides (e.g., Fe_3_O_4_ and γ‐Fe_2_O_3_),[[qv: 104a,d]] after surface functionalization^105^ (such as metal or metal oxide,[[qv: 105a]] SiO_2_,[[qv: 105b]] and surfactants).[[qv: 105c]] II) The use of magnetic hybrid composites with the MNPs as a magnetite core combined with an adsorbent shell. The core–shell architecture can efficiently impede the aggregation of magnetite cores and prevent loss of magnetism, while allowing feasible surface modification, enabling them to be a desired candidate for MSPE. To date, many magnetic hybrid adsorbents with core–shell nanostructure have been used in MSPE, such as MIPs,^106^ carbon nanotubes,[[qv: 104b,107]] graphene,[[qv: 104e,108]] MOFs,[[qv: 104c,109]] etc. However, the above functional magnetic adsorbents suffer from some drawbacks, such as tedious synthesis procedures, poor structural stability, or low selectivity. Against such a backdrop, the incorporation of the fascinating features of POFs and MNPs to construct a new category of magnetic hybrid adsorbents,^110^ with both enhanced functionality and magnetic separability, is of great significance.

**Figure 10:**
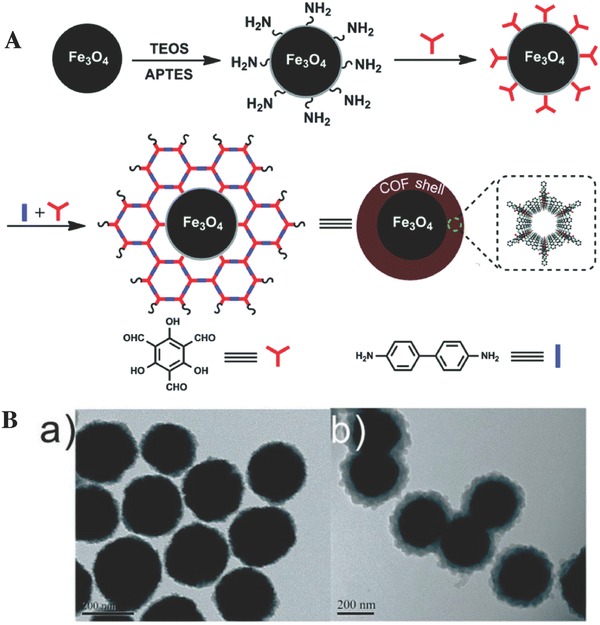
A) Preparation of core–shell Fe_3_O_4_@TpBd nanospheres using an in situ growth method. B) Transmission electron microscopy images of a) Fe_3_O_4_ MPs and b) Fe_3_O_4_@TpBd nanospheres. Adapted with permission.[[qv: 110b]] Copyright 2017, The Royal Society of Chemistry.

In 2016, Tan et al.[[qv: 110a]] pioneered a thermodynamically controlled method for translating amorphous polyimine networks into crystalline imine‐linked COFs (TpBd) on the surface of Fe_3_O_4_‐MNPs. This prepared core–shell Fe_3_O_4_@TpBd (*S*_BET_ = 1346 m^2^ g^−1^) exhibited a good photothermal conversion ability and displayed great potential in phototherapy. Yan and co‐workers[[qv: 110b]] subsequently presented a solvothermal in situ growth method for the construction of Fe_3_O_4_@TpBd nanospheres (*S*_BET_ = 272.6 m^2^ g^−1^), with a core–shell architecture for MSPE of bisphenols from water. As illustrated in **Figure** **10**A, the core–shell Fe_3_O_4_@TpBd was prepared using the following three steps: I) the Fe_3_O_4_ MNPs (TEMs in Figure 10B‐a), acted as the magnetic core and were modified by tetraethyl orthosilicate (TEOS) and APTES to form amino functionalized MNPs (Fe_3_O_4_‐NH_2_); II) Tp, a COF‐TpBd monomer, was grafted onto the surface of Fe_3_O_4_‐NH_2_ via Schiff base chemistry; III) Fe_3_O_4_‐NH_2_‐Tp was mixed with Tp and Bd solution in mesitylene/dioxane (1:1) to further complete the imine‐bonds formation between Tp and Bd for the synthesis of Fe_3_O_4_@TpBd (Figure 10B‐b) using acetic acid as catalyst. After changing the concentration of Tp and Bd monomers, the shell thickness of Fe_3_O_4_@TpBd was easily regulated. Furthermore, due to the interactions of π–π and hydrogen bonds between COF‐TpBd shell and analytes, these magnetic nanospheres can be utilized for MSPE of bisphenol A and bisphenol AF with rapid and high extraction capacity. Almost at the same time, Chen et al. prepared the magnetic Fe_3_O_4_@COF‐1[[qv: 110c]] and Fe_3_O_4_@COF‐LZU1[[qv: 110d]] for the MSPE of PAHs and paclitaxel from the environmental and biological samples. However, these above synthetic approaches usually require some harsh experimental conditions (e.g., elevated temperature and relative long reaction time), and many researchers are trying to find several more convenient approaches to prepare magnetic‐POFs for MSPE.

**Figure 11:**
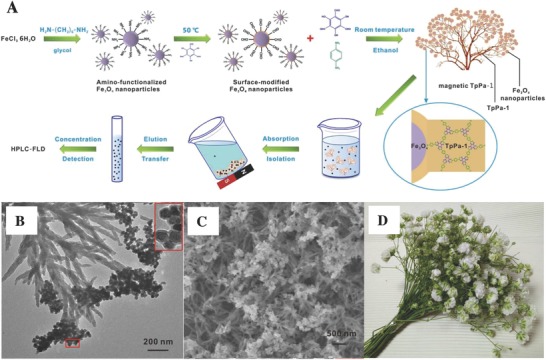
A) Schematic illustration of synthetic and MSPE procedures of bouquet‐shaped Fe_3_O_4_@TpPa‐1. B) TEM and C) SEM images of the magnetic Fe_3_O_4_@TpPa‐1. D) A picture of a gypsophila bouquet. Adapted with permission.^112^ Copyright 2017, American Chemical Society.

Liao and co‐workers^111^ reported to synthesize a series of MNPs, (Fe_3_O_4_, Co_3_O_4_, and NiO)@COF‐LZU1 through a simple two‐step method involving mechanochemical grinding and crystallization. Among them, Fe_3_O_4_@COF‐LZU1 (*S*_BET_ = 872 m^2^ g^−1^), with well‐defined pore channels and redox‐active properties, showed promising applications for the magnetically recoverable adsorbents and electrochemical energy storage. By grafting TpPa‐1 onto the surface‐modified Fe_3_O_4_, He et al.^112^ synthesized an interesting bouquet‐shaped magnetic Fe_3_O_4_@TpPa‐1 (*S*_BET_ = 247.8 m^2^ g^−1^) with core–shell structure using a facile room temperature approach. The schematic illustration of preparation and the MSPE process of Fe_3_O_4_@TpPa‐1 are displayed in **Figure** **11**. The bouquet‐like magnetic COFs were assembled together through covalent bonds, with Fe_3_O_4_@TpPa‐1 nanoparticles as the “flowers” and TpPa‐1 nanofibers as the “stems” (Figure 11B–D). Coupled with HPLC‐FLD detection, a MSPE method, based on the bouquet‐like Fe_3_O_4_@TpPa‐1, was developed for preconcentration of PAHs from water samples, with acceptable repeatability. Lin and co‐workers^113^ developed a rapid room temperature method to synthesize Fe_3_O_4_@TAPB‐TPA (*S*_BET_ = 181.36 m^2^ g^−1^) with a core–shell architecture using monodisperse Fe_3_O_4_ as magnetic core, with TAPB and TPA as the comonomers. With a high saturation magnetization (42.7 emu g^−1^), Fe_3_O_4_@TAPB‐TPA demonstrated an excellent MSPE adsorbent for the extraction of bisphenols. This group also reported the room temperature synthesis of Fe_3_O_4_@TbBd (*S*_BET_ = 196.21 m^2^ g^−1^)^114^ for the MSPE of some hydrophobic peptides from human serum samples. Fe_3_O_4_@TbBd showed a good selectivity to the hydrophobic peptides while indicating a size‐exclusion effect against macromolecules (e.g., proteins).

The above studies expanded the scope of magnetic‐COFs and exhibited the promising potential for biochemical analysis.^115^ The enrichment of glycopeptides[[qv: 115a,b]] and trypsin[[qv: 115c]] through magnetic‐COFs assisted MSPE has also been developed. For instance, Qian and co‐workers[[qv: 115a]] reported a two‐step solvothermal method to synthesis a magnetic Fe_3_O_4_@TpPa‐1 with a sea urchin structure for MSPE of *N*‐glycopeptides from human serum digests. This MSPE method exhibited a low LOD, satisfactory selectivity, and acceptable recovery toward *N*‐linked glycopeptides. A COF‐5 modified magnetic graphene biocomposite (MagG@COF‐5, *S*_BET_ = 201 m^2^ g^−1^) was synthesized by Wang et al.[[qv: 115b]] using a simple self‐assembly method for magnetic solid‐phase extraction of glycopeptides. This MagG@COF‐5 integrated the features of COF‐5 and the magnetic graphene (e.g., the hydrophilic properties, porous structure, and high surface area from COF‐5, as well as the delocalized π–π electron system from graphene). The as‐synthesized MagG@COF‐5 exhibited excellent performance in MSPE of *N*‐linked glycopeptide, with a low LOD (0.5 fmol µL^−1^), a size‐exclusion effect (HRP digests/BSA, 1:600), good recyclability, and reusability.

Using FeCl_3_·6H_2_O as an iron oxide precursor and ZnCl_2_ as a Lewis acid catalyst, Zhang and co‐workers^116^ reported the synthesis of Fe_2_O_3_/CTF‐1 (*S*_BET_ = 930–1149 m^2^ g^−1^) through a microwave‐enhanced high‐temperature ionothermal method. Their results showed that Fe_2_O_3_/CTF‐1 exhibited a good adsorption capacity (291 mg g^−1^) and a rapid adsorption kinetics (*k*_ads_ = 4.31 m^2^ mg^−1^ min^−1^), as well as a perfect MSPE performance for the methyl orange from aqueous solution. Recently, Hu and co‐workers^117^ synthesized a magnetic Ni/CTF‐1 composite (*S*_BET_ = 239 m^2^ g^−1^) through in situ reduction of nickel ions on CTF‐1 using a solvothermal method. A new Ni/CTF‐1‐assisted MSPE‐GC‐FID method was then established for the analysis of PAEs from various plastic packaging materials, with relative recoveries ranging from 70.6% to 119%. The same group^118^ also reported the synthesis of MOP‐2 (*S*_BET_ = 239 m^2^ g^−1^)[[qv: 118a]] and MOP‐SH (*S*_BET_ = 270 m^2^ g^−1^),[[qv: 118b]] with good performance for MPSE of dye pollutants or Hg(II) from water samples.

#### 3.3.4 Solid Phase Microextraction

SPME is a nonexhaustive sample pretreatment method which has been demonstrated to be well‐suited for sensitive and selective preconcentration of various analytes, particularly for volatile and semi‐volatile species in a plethora of studies.^119^ Introduced in the early 1990s, SPME addresses several challenges in traditional sample preparation methods, which successfully integrates many analytical steps into one step, such as sampling, preconcentration, as well as sample introduction for instrumental analysis. In this technique, the adsorption equilibrium is based on the fused silica or metal fiber substrate covered with an appropriate sorbent coating thin layer. Therefore, it is crucial to seek a suitable coating adsorbent that can bring high extraction efficiency and low limits of detection.[[qv: 90b,120]] Up to now, only limited coating materials are commercially available from Supelco (Sigma‐Aldrich, USA), including polydimethylsiloxane (PDMS), carboxen/polydimethylsiloxane (CAR/PDMS), polydimethylsiloxane/divinylbenzene (PDMS/DVB), polyacrylate (PA), etc.^121^ To surmount these limitations, recent advancements in SPME have concentrated on the design of new adsorbent materials with high mechanical and chemical stability. Since the creation and introduction of SPME, a great number of innovative materials^122^ have been applied to the preconcentration of diverse analytes from the multiple real samples, such as ionic liquids (ILs) or polymeric ionic liquids (PILs),[[qv: 122a]] carbon materials (carbon nanotubes (CNTs),[[qv: 122b,c]] graphene,[[qv: 122d,e]] and graphitic carbon nitride (g‐CN),[[qv: 122f,g]]) metal/metal oxide nanoparticles,[[qv: 122h,i]] molecularly imprinted polymers (MIPs),[[qv: 122j]] MOFs,[[qv: 122k–m]] etc.

**Figure 12:**
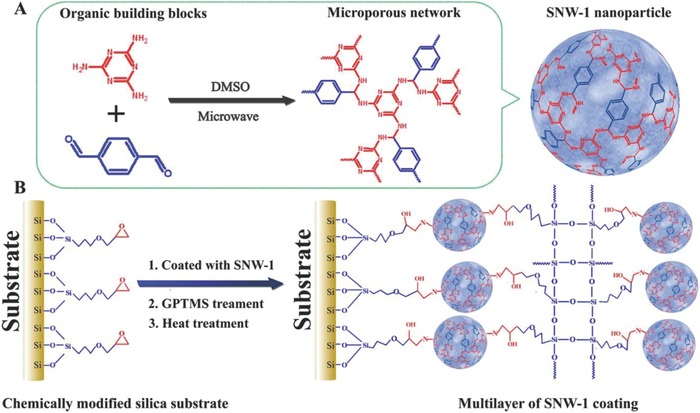
Schematic representations of the preparation of SNW‐1 using an A) microwave‐assisted method and B) the fabrication of the SNW‐1 coated SPME fiber. Adapted with permission.[[qv: 123a]] Copyright 2015. American Chemical Society.

Recently, POFs‐coated SPME fibers have been demonstrated for efficient enrichment of various analytes^123^ including PAHs,[[qv: 123a,b]] volatile fatty acids (VFAs),[[qv: 123a]] phenols,[[qv: 123c,d]] benzene homologues,[[qv: 123e,f]] styrene,[[qv: 123f]] pyrethroids,[[qv: 123g]] organochlorine pesticides (OCPs),[[qv: 123h,i]] antioxidants, and preservatives.[[qv: 123j]] Utilizations of POFs‐assisted SPME coatings for analytical chemistry are also summarized in Table 2. The first instance of using POFs as an SPME coating was introduced by Pan and co‐workers.[[qv: 123a]] In this approach, a multilayer interbridging procedure was employed to chemically bond a thin film of microwave‐synthesized SNW‐1 (*S*_BET_ = 231 m^2^ g^−1^) on the silica substrate to prepare SNW‐1 coated SPME fibers (**Figure** **12**). This SNW‐1 coated SPME fiber has an outstanding chemical stability in different organic solvents, as well as solutions. In addition, the SNW‐1 coating exhibited a higher enrichment performance of PAHs and VFAs over commercial PDMS and PDMS/DVB coatings via π–π and acid–base interactions. Furthermore, this SPME fiber was coupled with GC‐MS detection for the determination of VFAs from tobacco shred and tea leaf samples. Our group[[qv: 123c]] also prepared a SNW‐1 coated SPME fiber using a chemical cross‐bonding method by fabrication of the solvothermal synthesized SNW‐1 (*S*_BET_ = 668 m^2^ g^−1^) onto a silanol‐modified stainless steel wire. The developed method is based on SNW‐1 coated fiber and exhibited high enrichment factors, good linearity, low limits of detection, and acceptable repeatability for SPME of phenols from honey samples. Later, we synthesized an amide‐linked COF, COF‐SCU1 (*S*_BET_ = 65.3 m^2^ g^−1^),[[qv: 123e]] for the headspace SPME of volatile benzene homologues from indoor air as well as PAF‐6 (*S*_BET_ = 159 m^2^ g^−1^)[[qv: 123b]] for SPME of PAHs, phthalate plasticizers, and *n*‐alkanes. Jia and co‐workers contributed substantial works to prepare POFs‐assisted SPME coatings, such as hydrazine COFs,[[qv: 123g,h]] PAF‐1‐NH_2_/IL,[[qv: 123i]] and PAF from Chan–Lam reaction.[[qv: 123j]] For instance, a hydrazine‐linked COF[[qv: 123g]] was synthesized from the condensation of terephthalic dihydrazide and 1,3,5‐benzenetricarboxaldehyde, and this hydrazine COF was then utilized as the novel coatings in SPME of pyrethroids from vegetable and fruit samples with high enrichment factors.

SPME fiber coatings were also fabricated by combining COFs with ionic liquids[[qv: 123i]] or gel[[qv: 91,123f]] to further improve the extraction efficiency of COFs. An amine‐functionalized PAF‐1 and ionic liquid (PAF‐1‐NH_2_/IL) hybrid material was constructed by Jia and co‐workers[[qv: 123i]] and used for the SPME coating. Coupled with GC‐ECD detection, the PAFs/IL coated fiber was employed for the preconcentration of OCPs from milk and juice samples with the enhancement factors of 247–1696. Jin et al.[[qv: 123f]] reported the synthesis of a PAF‐48/gel hybrid coating for SPME of styrene and benzene homologues from liquid food simulants with a wide linearity, low limits of detection, good repeatability, and long reused times. The PAF‐48/gel coated SPME fiber showed an extraction selectivity for styrene and benzene homologues over other organic analytes and exhibited a molecular sieving effect for the molecules with different sizes. Similarly, Huang and co‐workers^124^ further studied the relationship between the pore structure and the adsorption efficiency of the POFs by synthesizing three POFs with similar properties but different pore volumes. Their results showed that the POFs with a higher pore volume achieved a better SPME efficiency than their counterparts with smaller pore volume, as analytes with a smaller size than the pore size of POFs might be more efficiently adsorbed into the pores.

## 4 Conclusions and Perspectives

This review outlined the state‐of‐the art on the preparation and utilization of POFs as the advanced materials in the fields of analytical chemistry. Their excellent features, such as diverse synthetic methods, controllable pore sizes, high surface areas, and good crystalline frameworks, indicated their outstanding potential applications for the fluorescence detection, chromatographic separation, as well as sample preparation. Not only the COFs, CTFs, and PAFs but also their functionalized composites have been constructed to meet the requirements of various techniques in analytical chemistry.

In the case of the fluorescence detection, a comprehensive literature has demonstrated that POFs are promising candidates for the detection of nitrobenzene derivatives, small molecules, and metal ions dependent on their excellent fluorescence properties. These fluorescent POFs can be constructed by choosing appropriate fluorescent‐groups or using suitable linkage mode to increase the conjugation systems of POFs resulting in an improved sensitivity. The modification of the POFs structures with specific functional groups can efficiently improve their selectivity (e.g., sulfur groups are attributed to the selective identity of Hg^2+^). As mentioned above, the fluorescence quenching (turn‐off) mode is frequently encountered in fluorescent chemical sensing of the electron‐withdrawing species; however, some analytes of rich‐electrons possessing higher‐lying LUMOs than the fluorescent POFs can also be detected by “turn‐on” fluorescence enhancement, based upon the AIE mechanism. Although few reports have been published on this subject, we believe that the “turn‐on” AIE‐POFs have great potential in fluorescence detection. Moreover, the detection range, accuracy, sensitivity, and selectivity of fluorescence‐based methods can be enhanced by synthesizing more types of POFs through the design of new structures using the innovative monomers.

In terms of chromatographic separation, POFs have shown great potential as the stationary phases in GC, HPLC, and CEC systems. Because of the molecular sieving effect, van der Waals, hydrogen bonds, π–π, or hydrophobic interactions, POFs‐assisted chromatographic methods have achieved baseline separations of many specific analytes. Notably, some positional isomers and chiral molecules can also be successfully resolved using chiral POFs in GC and HPLC. However, the utilization of POFs as stationary phases is still in its infancy (most related studies are published after 2014) and still encounters many limitations. One of the most common issues that must be addressed is due to the inherent features of sub‐micrometer sizes and broad size distributions of POFs, it is impossible to employ them independently as the stationary phases for chromatographic separations, particularly for HPLC. Nevertheless, POFs are incorporated into the structure of monolith or silica, which has been reported. There is also increasing interest in developing new techniques to incorporate POFs onto other porous supporting scaffolds for chromatographic separation. Furthermore, introducing the POFs onto the supporting materials allows them to have a more homogeneous surface that could be further modified with other functional groups, which might lead to the further development of a broad range of chromatographic columns showing enhanced selectivity.

In addition, the applications of POFs for sample preparation has become a gradually developed field, and an increasing number of COFs, CTFs, and PAFs, along with their functional composites, have been designed and utilized for SPE, D‐µ‐SPE, MSPE, SPME, and other novel techniques. Thus, there has been a trend that POFs might replace other conventional sorbents which have been typically applied for sample preparation. Even so, the development of POFs in sample pretreatment methods is still faced with some barriers to enhance their extensive application. The primary challenge is that POFs employed in this field are currently limited to some easily synthesized COFs, CTFs, or PAFs. For instance, only a few boronate‐, Tp‐, or imine‐linked COFs, such as COF‐5, TpPa‐1, TpPa‐2, TpBd, SNW‐1, LZU1, etc., have been developed for sample preparation. In the series of CTFs, only the ionothermal‐synthesized CTF‐1 has been successfully used in analytical research. PAF‐1, PAF‐6, and PAF‐48 utilized in sample pretreatment methods only represent a small proportion of diverse PAFs. The future applications of COFs, CTFs, or PAFs as sorbents in sample preparation need further research on the construction of new POFs with higher chemical stability, enhanced selectivity, super adsorption features, more‐effective cost reduction, and better reusability.

Fortunately, the increasing amount of new POFs with fascinating features vastly increases the possibility of their applications in analytical chemistry. These innovative POFs originated from different building blocks and diverse architectures might be fabricated in a controlled manner, providing a good method to design and synthesize the POFs matched for specific analytical chemistry field. Given the potential of COFs, CTFs, or PAFs and the demonstrated success in published studies, we believe that more POFs and their functional composites will be increasingly prepared and utilized in analytical chemistry, along with the progressive synthetic techniques. Furthermore, in the near future, specific functional POFs could be designed for specific analytical chemistry applications.

## Conflict of Interest

The authors declare no conflict of interest.
